# Imbalance of helper T cell type 1, helper T cell type 2 and associated cytokines in patients with systemic lupus erythematosus: A meta-analysis

**DOI:** 10.3389/fphar.2022.988512

**Published:** 2022-09-29

**Authors:** Shate Xiang, Jingjing Zhang, Mengge Zhang, Suhai Qian, Rongyun Wang, Yao Wang, Yingshi Xiang, Xinghong Ding

**Affiliations:** ^1^ School of Basic Medical Sciences, Zhejiang Chinese Medical University, Hangzhou, China; ^2^ First Clinical School of Medicine, Nanjing Medical University, Nanjing, China

**Keywords:** systemic lupus erythematosus, helper T cell type 1, helper T cell type 2, cytokines, meta-analysis

## Abstract

**Objective:** Th1 and Th2 cells and their associated cytokines function in the pathogenesis of systemic lupus erythematosus (SLE), but their exact roles are uncertain. We performed a meta-analysis to examine the relationship of these cells and cytokines with SLE.

**Methods:** Multiple databases were searched to identify publications that reported the percentages of Th1 and Th2 cells and their associated cytokines in SLE patients and healthy controls (HCs). Meta-analysis was performed using Stata MP version 16.

**Results:** SLE patients had a lower percentage of Th1 cells, a higher percentage of Th2 cells, and higher levels of Th1- and Th2-associated cytokines than HCs. SLE treatments normalized some but not all of these indicators. For studies in which the proportion of females was less than 94%, the percentage of Th2 cells and the level of IL-10 were higher in patients than HCs. SLE patients who had abnormal kidney function and were younger than 30 years old had a higher proportion of Th1 cells than HCs. SLE patients more than 30 years old had a higher level of IL-6 than HCs.

**Conclusion:** Medications appeared to restore the balance of Th1 cells and other disease indicators in patients with SLE. Gender and age affected the levels of Th1 and Th2 cells, and the abnormally elevated levels of Th2 cells appear to be more pronounced in older patients and males.

**Systematic Review Registration:** [https://www.crd.york.ac.uk/prospero/], identifier [CRD42022296540].

## Highlights

1) Compared with healthy people, patients with systemic lupus erythematosus have lower levels of Th1 cells and higher levels of Th2 cells.
2) The main effect of conventional medications is to increase the level of Th1 cells in SLE patients.
3) Older patients and men appear to have more abnormally elevated Th2 cell levels.

## Introduction

Systemic lupus erythematosus (SLE) is an inflammatory autoimmune disease characterized by the production of various autoantibodies and disruption of multiple organs and organ systems due to immunomodulatory dysfunction. The incidence of SLE is about 9 times higher in females than males (Yen and Singh, 2018). There is an incomplete understanding of the etiology of SLE, although evidence indicates that abnormal immune responses, B cell hyperactivation, and T cell abnormalities contribute to the pathogenesis (Catalina et al., 2020). For instance, a T cell autoimmune disorder leads to autoantibody production, and the production of several autoreactive T cells is an important precursor to B cell hyperactivation and the formation of immunoglobulins and immune complexes. Therefore, overactivation of T cells appears to have a major role in the pathogenesis of SLE (Katsuyama et al., 2018).

After antigen stimulation, immature CD4+T cells produce cytokines, T helper cells (Th1, Th2, and Th17) and regulatory T cells (Tregs) that have different immune functions (Zhu et al., 2010). The abnormal gene expression profiles of these cells are considered closely related to SLE (Wahren-Herlenius and Dörner, 2013; Talaat et al., 2015), and the balance of Th1/Th2 cells regulates the pathogenesis of SLE (Dolff et al., 2011). In particular, an imbalance of the pro-inflammatory effects of Th1 and the anti-inflammatory effects of Th2 is critical to immune dysfunction and infiltration of target organs by inflammatory cells (Moulton and Tsokos, 2011; Tsokos et al., 2016). Although many studies have examined the levels of Th1 and Th2 cells in patients with SLE, many of these results are discordant (Viallard et al., 1999; Amit et al., 2000; Li et al., 2002a; Oster et al., 2019). Thus, examination of changes in the levels of Th1 and Th2 cells during SLE is an active area of research (Matia-Garcia et al., 2021).

Different Th cells produce different cytokines. In particular, Th1 cells produce interferon (IFN)- γ, interleukin (IL)-2, and tumor necrosis factor (TNF)-α, whereas Th2 cells produce IL-4, IL-5, IL-6, and IL-10 (Muhammad Yusoff et al., 2020). Studies of the cytokine profiles of SLE patients indicated that dysregulated production of cytokines and soluble mediators were the primary factors responsible for increased inflammation (Lourenco and Cava, 2009). In particular, during SLE there is an increase in multiple cytokines, most of which have pro-inflammatory effects, although some of these cytokines have immunomodulatory and anti-inflammatory effects (Idborg and Oke, 2021). Because a variety of cytokines are associated with SLE disease activity, these cytokines were thought to be ideal biomarkers for defining SLE and as therapeutic targets for treatment of active SLE (La Cava, 2010; Rönnblom and Leonard, 2019; Aragón et al., 2020). Nevertheless, studies that examined changes of cytokines in the serum of SLE patients have reported contradictory results. For example, some studies reported increased levels of IL-4 (Davis et al., 2011), some studies reported no changes of IL-4 (Charles et al., 2010), and some studies reported reduced levels of IL-4 (Cavalcanti et al., 2017). Moreover, the effect of different factors on the regulation of cytokines during SLE is an active area of research. Although a few studies suggested that the effects of IL-4 may depend on genetic background, and that IL-2 function appears related to a sex-determined immune response, these conclusions remain uncertain (Moulton et al., 2012; Radmanesh et al., 2020).

In summary, there are many controversies in the research on the imbalance of Th1, Th2 and relevant cytokines in SLE patients. However, no evidence-based research has yet established the reasons for the inconsistent results of previous studies. Difference in the characteristics of patients (e.g., geographic region, gender, disease activity, medication use) may have contributed to the inconsistent results from previous studies. Therefore, this study aims to clarify the changes of Th1 cells, Th2 cells, and their related cytokines during SLE through meta-analysis, to better understand the pathogenesis of this disease, improve clinical diagnosis, and develop therapeutic strategies that have high efficacy and low toxicity.

## Methods

This systematic review was registered under the number CRD42022296540 in the International Prospective Register of Systematic Reviews (PROSPERO).

### Search strategy

Two researchers (M Zhang and J Zhang) independently searched the databases for publications from their dates of establishment to 30 September 2021. Search strategy was detailed in Supplementary file.

### Study selection criteria

All included studies were case-control studies; examined a population as the study object; and assessed the percentages of Th1 cells and Th2 cells or the concentrations of their associated cytokines in peripheral blood. Studies were excluded if they were animal experiments; contained irrelevant research content, were literature reviews, or were conference abstracts not published in full; had missing data; examined cytokines secreted by non-CD4^+^ cells (such as IL-2, IL-17, and IL-6 secreted by Tregs); or did not measure the levels of cytokines in whole peripheral blood.

### Data extraction and transformation

Two independent researchers (S Xiang and S Qian) extracted and cross-verified data necessary for analysis from eligible literature. Disagreements were resolved through discussion and consultation with a third researcher (Y Wang). The contents included: 1) the first author, the year of publication, the characteristics of subjects and country in which the study was performed; 2) The characteristics of included studies (number of patients, proportion of women, average age, average duration of disease, therapeutic drugs); 3) experimental methods (diagnostic criteria, determination methods); 4) Th1, Th2 and cytokine levels secreted by SLE patients and HCs.

All original data, including graphs and plots, were extracted for use in the meta-analysis. Because some publications only provided relevant data as graphs, GetData Graph Digitizer version 2.25 (http://getdata-graph-digitizer.com/) was used to extract these data (Liao et al., 2015; Tang et al., 2015; Shen et al., 2021). When studies provided medians and ranges (or interquartile ranges) instead of means and standard deviations, data conversion was performed using the method proposed by Xiang Wan (Wan et al., 2014). The Newcastle-Ottawa Quality (NOS) (Stang, 2010) was used to assess the quality of the included studies.

### Statistical analysis

Stata version 16.0 was used to perform statistical analyses. When data were continuous variables and the measurement methods were similar, weighted mean differences (WMDs) were used as the effect scale. When the studies evaluated similar results using different measurement methods, or there was a large difference in the means or standard deviations, then standardized mean differences (SMDs) were used as the effect scale (Bakbergenuly et al., 2020). I^2^ was used to evaluate heterogeneity, and values of 25, 50, and 75% were used for classification of low, moderate, and high heterogeneity, respectively (Higgins et al., 2003). The meta-analysis was performed using a random-effects model that was weighted according to the sample size of studies (Wu et al., 2021). A difference with a *p* value below 0.05 was considered statistically significant. When the heterogeneity was high (I^2^ > 75%), subgroup analysis or sensitivity analysis (deleting one publication at a time) was used to assess the source of heterogeneity. Publication bias was evaluated using the Egger test (Hayashino et al., 2005), Begg test (Jiang et al., 2019), and a funnel plot.

## Results

### Study selection and characteristics

We retrieved 5,463 potentially eligible studies from the databases, and 4,601 studies remained after removal of duplicates. Screening of the titles and abstracts led to removal of 4,076 studies because the research content was considered irrelevant. Reading the full text of the remaining studies led to removal of 447 studies because they did not meet the eligibility criteria. Review of the remaining studies led to removal of 1 study that used different diagnostic criteria for SLE, 1 study that reported Th1 and Th2 cell levels in absolute numbers (cells/μL), and 38 studies that had research goals considered irrelevant to our meta-analysis. Further analysis of the 38 remaining studies indicated that 6 studies were ineligible because of insufficient quality and 6 studies were ineligible because of incomplete data. We finally included 26 studies in the meta-analysis (Supplementary Figure S1, Table 1). All 26 studies reported samples that were obtained from human peripheral blood, and used IFN-γ as a marker for Th1 cells and IL-4 as a marker for Th2 cells. The NOS score of each study was between 6 and 9 (Supplementary Table S1).

**TABLE 1:** The characteristics of included studies.

Study	Location	SLE Case/HC Case			SLE Case	Experimental methods
	Country	Number (female Sex/%)	Age (mean ± SD/Mean)	An Average Duration of Disease (years)	Medication	Technique
Feng Han Han et al. (2004) (2004)	China	30 (100%)/20 (100%)	28 ± 7/28 ± 5	2.8 ± 2	—	Flow cytometry
Xiaojuan Liu Liu and Qian, (2005) (2005)	China	35 (97.1%)/20 (90%)	28	3	Have not used glucocorticoids, immunosuppressants and vasodilators	Flow cytometry
Weijia Xu Xu et al. (2013) (2013)	China	38 (89.5%)/20 (85%)	40 ± 13/40 ± 11	5.0 ± 4.8	—	Flow cytometry
Yong Wang Wang et al. (2006) (2006)	China	18 (94.4%)/15 (86.7%)	31.5 ± 14.1/28.6 ± 10.3	Newly diagnosed	NA	Flow cytometry
Li Li et al. (2002b) (2002)	China	35 (94.3%)/10 (90%)	36.2/33.7	Newly diagnosed	NA	Flow cytometry
Shaoran Zhang Zhang, (2011) (2011)	China	89 (91%)/27 (88.9%) —	32.0 ± 11.9/31.4 ± 12.5	—	Prednisone, cyclophosphamide, vincristine, hydroxychloroquine	Flow cytometry
Xiaodong Wang (Wang et al., 2002) (2002)	China	35 (97.1%)/10 (80%) —	35.6/30.1	Newly diagnosed	NA	Flow cytometry
Xu-yan Yang Yang et al. (2013) (2013)	China	65 (89.2%)/30 (83.3%)	34 ± 11/32 ± 10	1.5 ± 1.2	Active SLE (NA); Inactive SLE (prednisone, hydroxychloroquine, azathioprine)	Flow cytometry
Yanni Jiang (Matia-Garcia et al. (2021) (2021)	China	97 (28.9%)/50 (38%)	35.4 ± 6.3/36.0 ± 8.4	—	—	Flow cytometry
Yufeng Yang Yang, (2015) (2012)	China	103 (86.4%)/23 (82.6%)	35 ± 13/38 ± 10	—	—	Flow cytometry
Roba M. Talaat (Talaat et al. (2015) (2015)	Egypt	60 (93.3%)/24 (91.7%)	28.6 ± 7.3/29.7 ± 7.0	5.0 ± 3.4	Glucocorticoids, antimalarial, azathioprine, cyclophosphamide, cyclosporine	Enzyme-linked immunosorbent assay
Diana C (Salazar-Camarena et al. (2019) (2019)	Mexico	36 (NA)/15 (NA)	32/35	6.7	Prednisone, azathioprine, antimalarials	Luminex xMAP
A. Cavalcanti Cavalcanti et al. (2017) (2017)	Brazil	51 (92%)/47 (91%)	15/15	3	Hydroxychloroquine, prednisone, mycophenolate mofetil, azathioprine, methotrexate	Flow cytometry
Diana Go’ mez (Gómez et al., 2004) (2004)	Colombia	51 (98.1%)/25 (NA)	34.2 ± 12.6/34.2 ± 5	4.9 ± 7.6	Prednisolone, cyclophosphamide, azathioprine, chloroquine	Enzyme-linked immunosorbent assay
PA´L SOLTE´SZ Lourenco and Cava, (2009) (2002)	Hungary	8 (87.5%)/19 (63.2%)	33/30.7 ± 5.5	3.5	Methylprednisolone, cyclophosphamide, azathioprine, hydroxychloroquine	Enzyme-linked immunosorbent assay
Pablo Medrano-Campillo Medrano-Campillo et al. (2015) (2015)	Spain	20 (100%)/20 (100%)	41.6 ± 9.7/41.6 ± 9.4	6.5 ± 3.5	Hydroxychloroquine, non-steroidal anti-inflammatory drugs	Flow cytometry
Lorena Álvarez-Rodríguez Álvarez-Rodríguez et al. (2019) (2019)	Spain	11 (100%)/21 (71.4%)	32.8 ± 13.1/40.3 ± 11.6	—	Antiaggregant, anticoagulant, corticosteroids, antimalarials	Flow cytometry
Daniel J Perry Perry et al. (2020) (2020)	America	39 (100%)/23 (100%)	45.8 ± 10.8/30.9 ± 8	—	Hydroxychloroquine, mycophenolate, mofetil, prednisone, methotrexate, azathioprine	Microarrays and NanoString assays
Katherine A Murphy Murphy et al. (2019) (2019)	America	5 (100%)/5 (100%)	32 ± 13.6/39 ± 6.9	11.8 ± 10.4	Hydroxychloroquine, mycophenolate mofetil	Flow cytometry
Weronika Kleczynska Kleczynska et al. (2011) (2011)	Poland	15 (93.3%)/11 (73%)	41.5 ± 13.8/34.0 ± 10.2	9.77 ± 5.69	Glucocorticoids	Flow cytometry
Sebastian Dolff Dolff et al. (2011) (2011)	Netherlands	24 (91.7%)/14 (85.7%)	41 ± 13/39 ± 12	—	Prednisone, azathioprine, mycophenolate mofetil, methotrexate, hydroxychloroquine	Flow cytometry
Mariana Postal Postal et al. (2013) (2013)	Brazil	57 (95%)/59 (91.2%)	18 ± 6.8/19 ± 6	4 ± 6.5	Prednisone, hydroxychloroquine, immunosuppressive drugs, azathioprine, cyclophosphamide, cyclosporine, methotrexate, mycophenolate mofetil	Enzyme-linked immunosorbent assay
C K Wong Wong et al. (2020) (2000)	China	36 (88.9%)/18 (88.9%)	35.9 ± 9/35.2 ± 7.5	6.8 ± 5.9	Prednisolone, hydroxychloroquine, azathioprine, cyclosporin	Enzyme-linked immunosorbent assay
Ding-lei SU Su et al. (2006) (2006)	China	41 (100%)/22 (100%)	35 ± 12/32 ± 7	—	NA	Enzyme-linked immunosorbent assay
Yan-bin Zhou Zhou et al. (2009) (2009)	China	10 (80%)/10 (80%)	26 ± 5/25 ± 6	1.3 ± 1.5	Not been treated with either glucocorticoids or immunosuppressants for at least 3 months prior	Flow cytometry
LCW Lit Lit et al. (2006) (2006)	Chia	80 (97.5%)/40 (97.5%)	36 ± 8/38 ± 9	12.1 ± 6.4	Prednisolone, hydroxychloroquine, azathioprine	Enzyme-linked immunosorbent assay

SLE, systemic lupus erythematosus; HC, healthy controls; SD, standard Deviation; NA, received no medication treatment missing data.

### Changes in the percentage of Th1 and Th2 cells in SLE patients

#### Th1 cells

**FIGURE 1:**
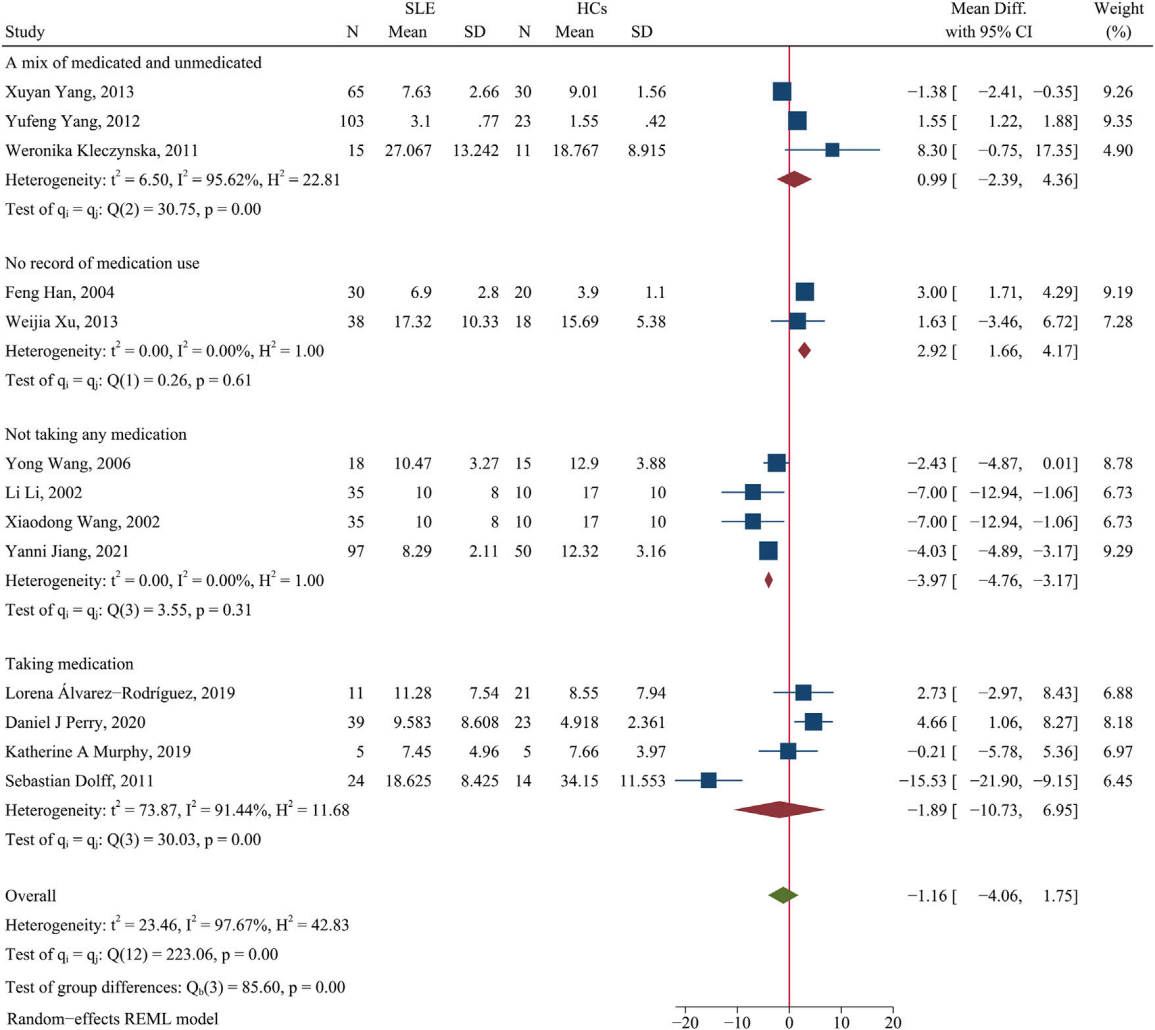
Forest plot of the percentage change of Th1 cells in SLE patients compared with HCs. The overall results (bottom) show the meta-analysis of 13 studies, and the other four results show meta-analyses of different subgroups according to medication use. SLE, systemic lupus erythematosus; HCs, healthy controls.

We found no significant difference in the percentage of Th1 cells in patients and HCs (WMD = −1.16; 95% CI = −4.06, 1.75; *p* = 0.43; n = 13; Figure 1), although there was high heterogeneity in these results (I^2^ = 97.67%).

We performed a subgroup analysis to assess the effect of medication use on Th1 cells. Studies that did not report medication use were categorized as an unrecorded medication group, and patients in other studies who were un-medicated or medicated were categorized into separate groups. The results showed that the percentage of Th1 cells was lower in unmedicated patients than HCs (WMD = −3.97; 95% CI = −4.76, −3.17; *p* < 0.001; n = 4; Figure 1). This implies that the use of medications by SLE patients affected the levels of their Th1 cells.

In total, 45 patients were unmedicated in the Xuyan Yang et al. study (Yang et al., 2013), and this accounted for 69% of their patients; their results showed that the percentage of Th1 cells was lower in patients than HCs (WMD = −1.38, 95% CI = −2.41, −0.35). A total of 41% of patients in the Yufeng Yang et al. study (Yang, 2015) were unmedicated; their results showed the level of Th1 cells was higher in patients than HCs (WMD = 1.55, 95% CI = 1.22, 1.88). Only 33% of patients in the Weronika Kleczynska et al. study (Kleczynska et al., 2011) were unmedicated; their results showed a trend for a higher level of Th1 cells in patients than HCs (WMD = 8.3, 95% CI = −0.75, 17.35). These results also suggest that SLE patients taking medications had a higher percentage of Th1 cells than HCs.

In addition, none of the patients in the Katherine A. Murphy et al. study (Murphy et al., 2019) used glucocorticoids (GCs), and their patients and HCs had similar percentages of Th1 cells. In contrast, all patients in the Sebastian Dolff et al. study (Dolff et al., 2011) used low-dose GCs, and their patients and HCs had very different percentages of Th1 cells. We speculate that use of low-dose GCs by SLE patients may alter the level of Th1 cells.

#### Th2 cells

**FIGURE 2:**
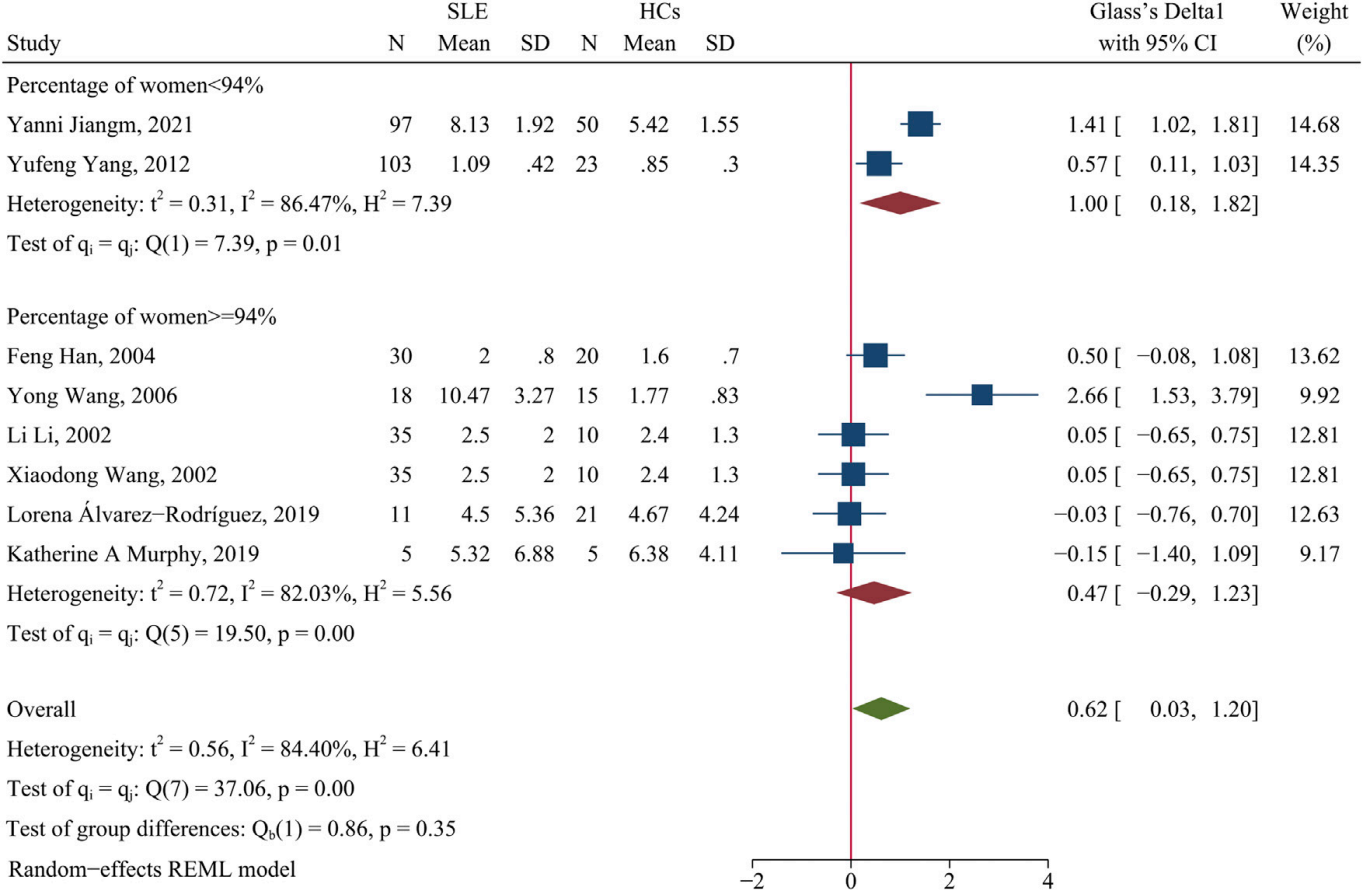
Forest plot of the percentage change of Th2 cells in SLE patients compared with HCs. The overall results (bottom) show the meta-analysis of 8 studies, and the other two results show meta-analyses of subgroups according to the percentage of females. SLE, systemic lupus erythematosus; HCs, healthy controls.

The meta-analysis showed there was a higher percentage of Th2 cells in patients than HCs (SMD = 0.62; 95% CI = 0.03, 1.20; *p* = 0.04; n = 8; Figure 2). Sensitivity analysis that excluded the Yong Wang study (Wang et al., 2006) and the Yanni Jiang study (Matia-Garcia et al., 2021) reduced the heterogeneity of these results (I^2^ = 2.22%; Supplementary Figure S2), and the percentage of Th2 cells remained higher in patients than HCs (SMD = 0.29; 95% CI = 0.03, 0.56; *p* = 0.03; n = 6).

Because the incidence of SLE is higher in women than men, we performed a subgroup analysis to analyze the effect of the proportion of women in each study on the outcome. When the proportion of women was at least 94%, SLE patients had a greater change in the percentage of Th2 cells than HCs (*p* > 0.05, Figure 2); however, even when the proportion of women was less than 94%, SLE patients had a significantly greater level of Th2 cells than HCs (SMD = 1.00; 95% CI = 0.18, 1.82; *p* = 0.02; n = 2; Figure 2). Subgroup analysis that excluded the Yong Wang study (Wang et al., 2006) reduced the heterogeneity of the studies in which the proportion of women was greater than 94% (Supplementary Figure S3), but the overall results did not change. Thus, for studies in which the proportion of women was less than 94%, the significant difference in the proportions of men and women may explain the high heterogeneity of the results.

The two studies in which the proportion of women was less than 94% were the Yufeng Yang et al. study (Yang, 2015) (86.4% women) and the Yanni Jiang et al. study (Matia-Garcia et al., 2021) (28.9% women), and both of these studies reported a higher percentage of Th2 cells in patients than HCs (Figure 2). In addition, a subgroup analysis indicated that medicated patients had a higher percentage of Th2 cells than HCs (SMD = 0.39; 95%CI = 0.08, 0.71; *p* = 0.01; n = 4; Supplementary Figure S4).

#### Disease activity and levels of Th1 and Th2 cells

We then compared the percentage of Th1 and Th2 cells in patients with active disease and inactive disease. The meta-analysis indicated disease activity had no significant relationship with the levels of Th1 or Th2 cells (*p* > 0.05, Supplementary Figures S5, S6).

#### Abnormal kidney function and levels of Th1 and Th2 cells

**FIGURE 3:**
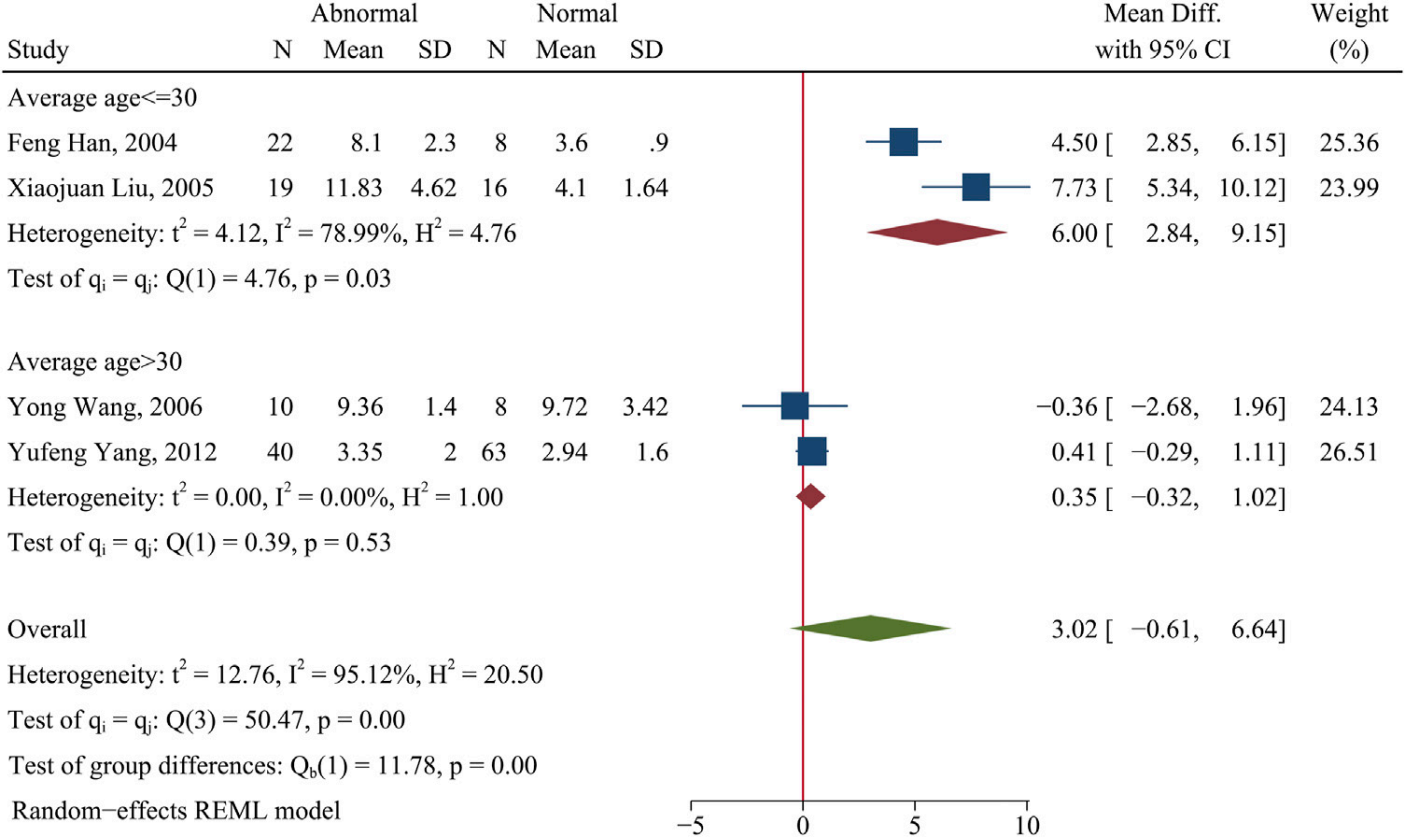
Forest plot of the percentage change of Th1 cells in SLE patients with abnormal kidney function compared with normal kidney function. The overall results (bottom) show the meta-analysis of 4 studies, and the other two results show meta-analyses of subgroups according to patient age. Abnormal: SLE patients with abnormal kidney function. Normal: SLE patients with normal kidney function.

The percentage of Th1 cells and Th2 cells were not obviously different in patients with abnormal kidney function (lupus nephritis or clinical presentation with proteinuria) and patients with normal kidney function (*p* > 0.05; Figure 3 and Supplementary Figure S7).

However, subgroup analysis indicated that age significantly affected the percentage of Th1 cells in patients with abnormal kidney function. In particular, patients who were 30 years old or less had a higher level of Th1 cells than HCs (WMD = 6.00; 95%CI = 2.84, 9.15; *p* = 0.0002; n = 2; Figure 3), but there was no significant difference in patients more than 30 years old (*p* > 0.05; Figure 3).

#### Changes of the Th1/Th2 ratio in patients with SLE

**FIGURE 4:**
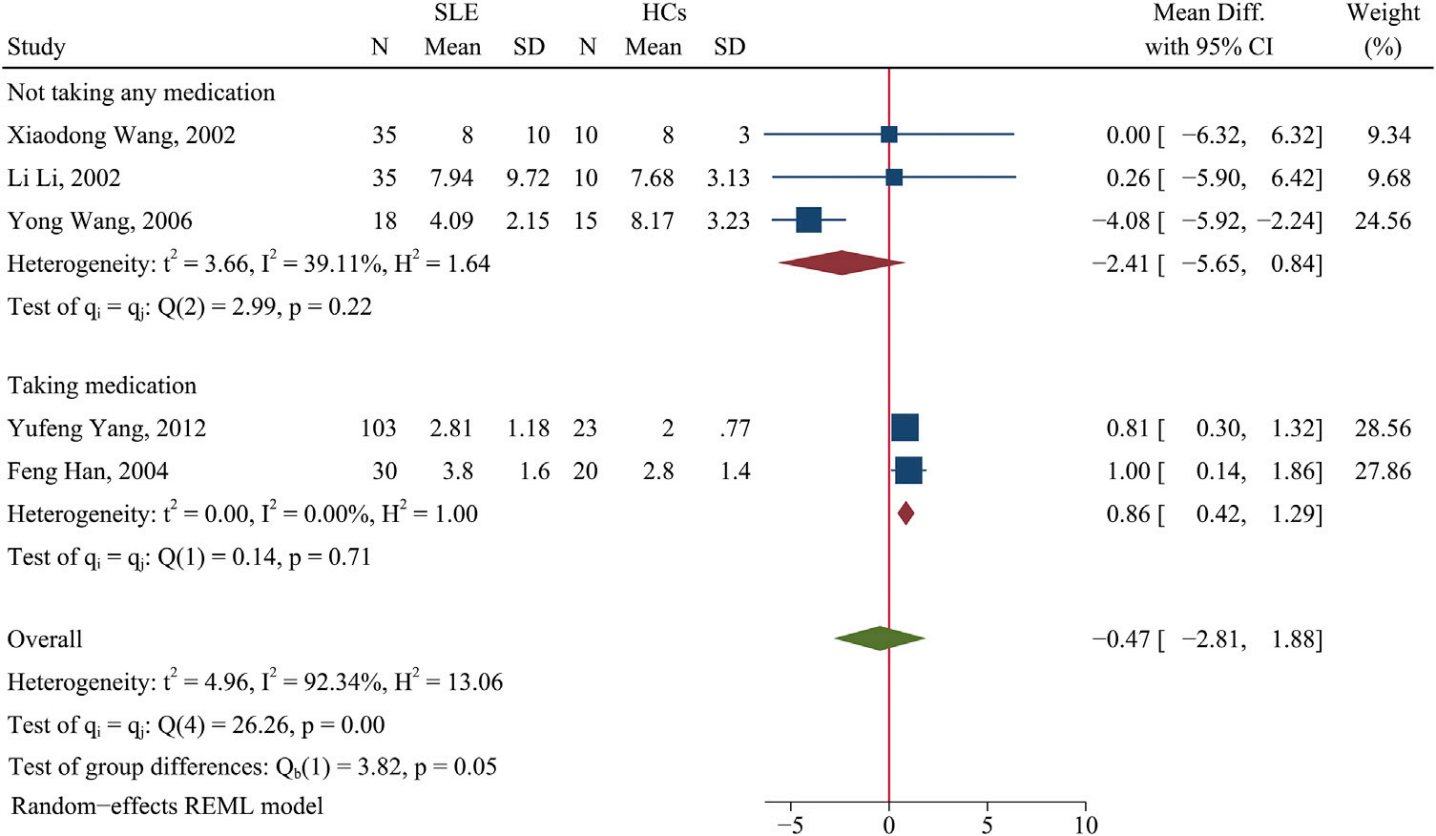
Forest plot of the ratio change of Th1/Th2 cells in SLE patients compared with HCs. The overall results (bottom) show the meta-analysis of 5 studies, and the two other results show meta-analyses of subgroups according to medication use. SLE, systemic lupus erythematosus; HCs, healthy controls.

There was no significant difference in the Th1/Th2 ratio of patients and HCs (*p* > 0.05; Figure 4). A subgroup analysis that assessed the effect of medication use showed that medicated patients had a higher Th1/Th2 ratio than HCs (WMD = 0.86; 95% CI = 0.42, 1.29; *p* < 0.001; n = 2; Figure 4), but there was no such relationship for unmedicated patients.

### Variation of Th1 and Th2 cytokines in patients with SLE

#### Th1 cytokines

**FIGURE 5:**
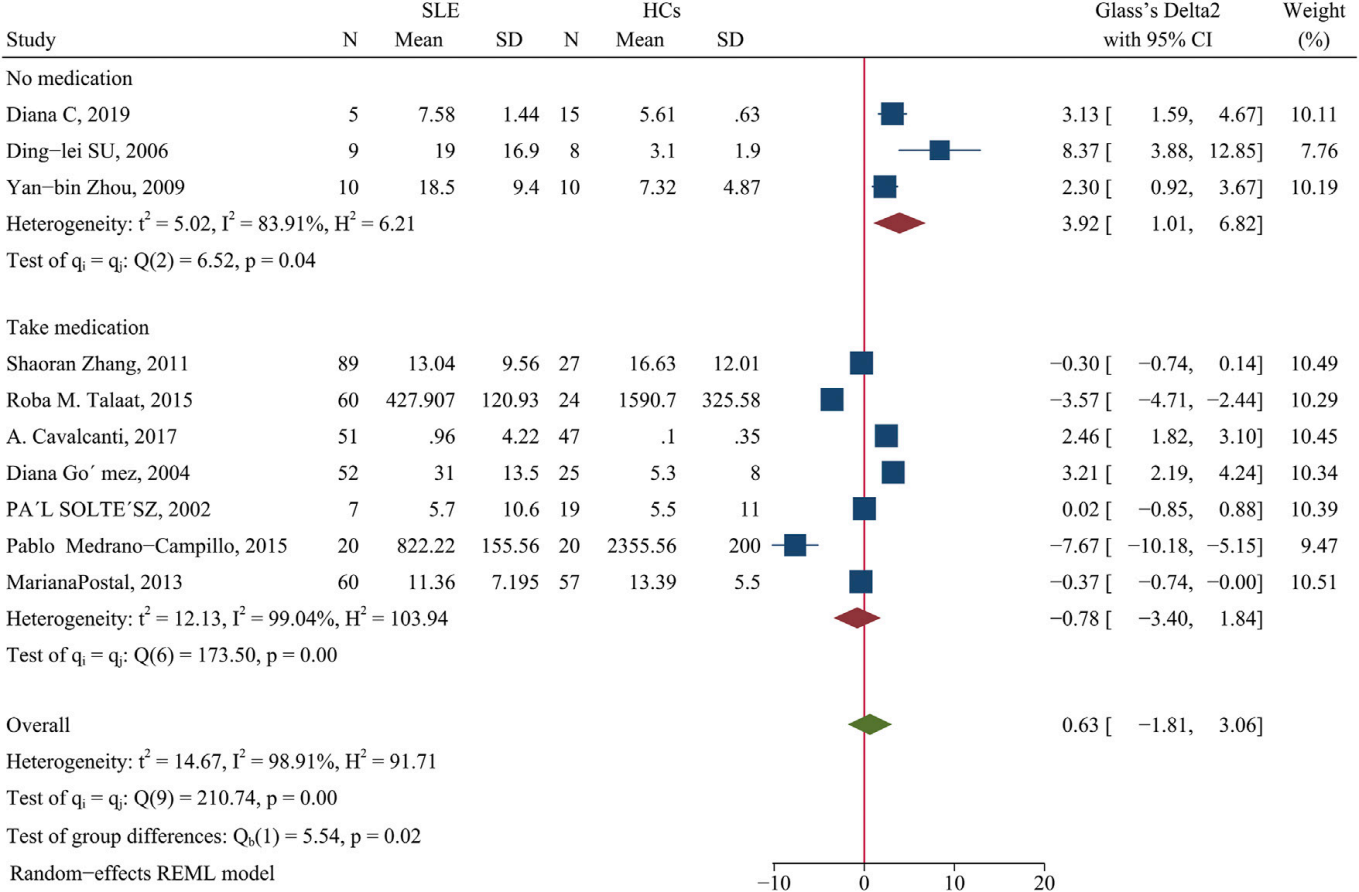
Forest plot of the concentration change of IFN-γ in SLE patients compared with HCs. The overall results (bottom) show the meta-analysis of 10 studies, and the other two results show meta-analyses of subgroups according to medication use. SLE, systemic lupus erythematosus; HCs, healthy controls.

Patients and HCs had no significant difference in the level of IFN-γ (SMD = 0.63; 95% CI = −1.81, 3.06; *p* = 0.61; n = 10; Figure 5). Subgroup analysis that examined the effect of medication use showed that unmedicated patients had a higher level of IFN-γ than HCs (SMD = 3.92; 95% CI = 1.01, 6.82; *p* = 0.008; n = 3; Figure 5), although there was no such difference in a comparison of medicated patients and HCs. This result suggests that unmedicated SLE patients have a higher level of IFN-γ than HCs, but medications normalize the level of IFN-γ in these patients.

**FIGURE 6:**
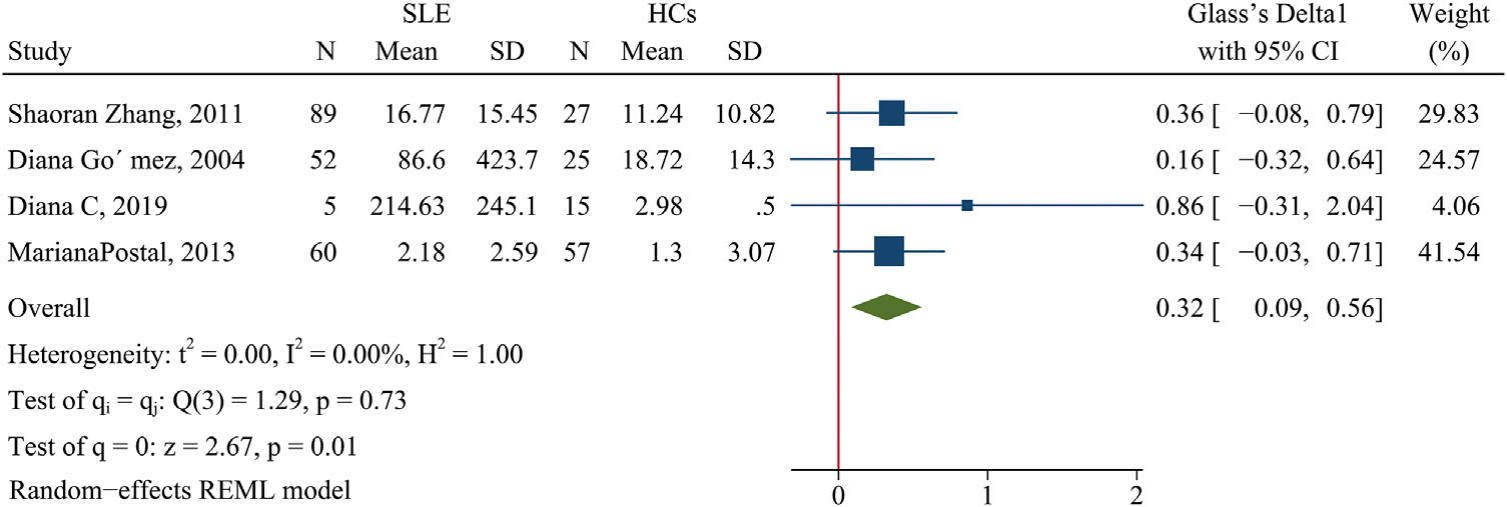
Forest plot of the concentration change of TNF-α in SLE patients compared with HCs. SLE, systemic lupus erythematosus; HCs, healthy controls.

The concentration of TNF-α was higher in patients than HCs (SMD = 0.32; 95% CI = 0.09, 0.56; P = 0.01; n = 4; Figure 6)

**FIGURE 7:**
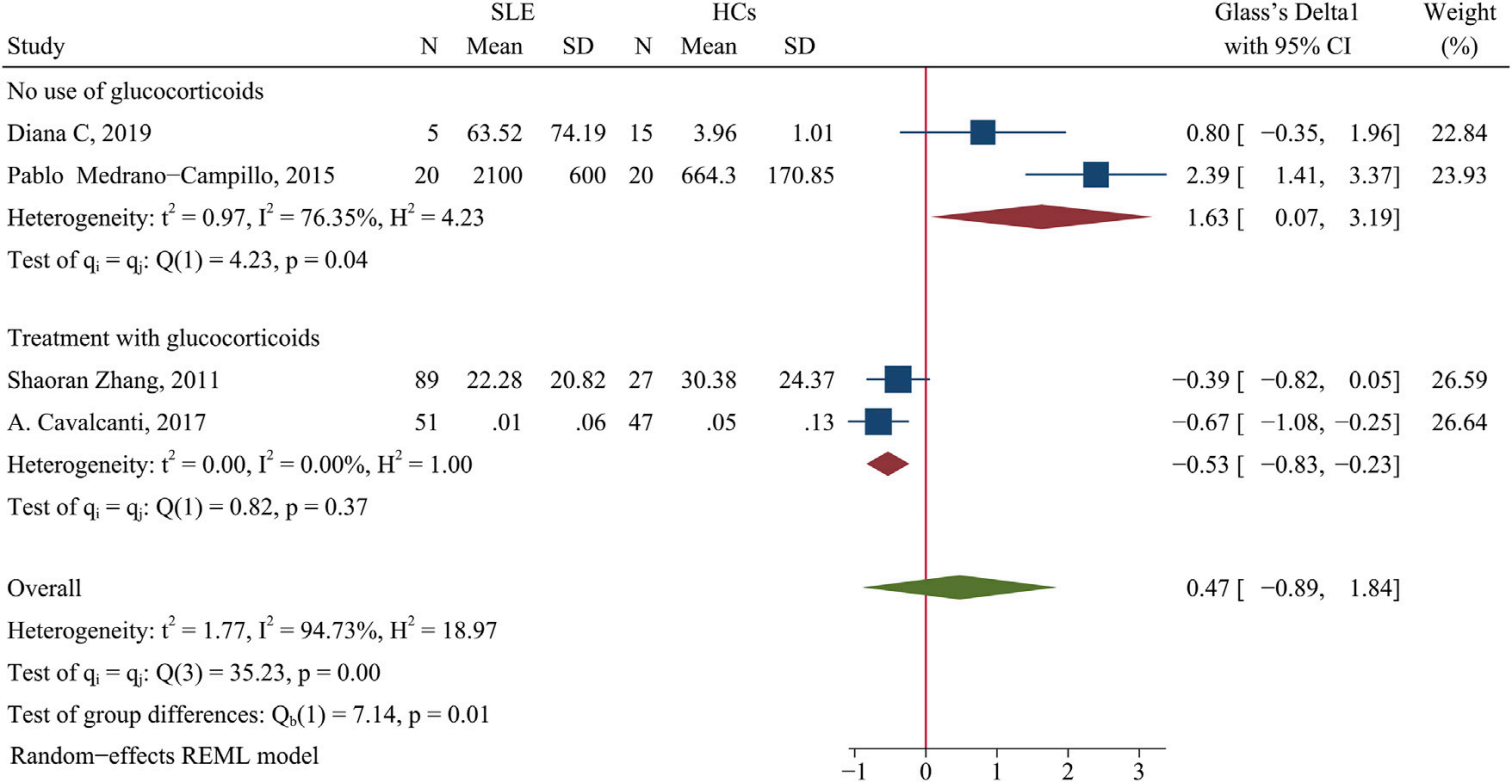
Forest plot of the concentration change of IL-2 in SLE patients compared with HCs. The overall results (bottom) show the meta-analysis of 4 studies, and the other two results show meta-analyses of two subgroups according to glucocorticoid use. SLE, systemic lupus erythematosus; HCs, healthy controls.

A meta-analysis indicated no significant difference in the IL-2 concentration of patients and HCs (SMD = 0.47; 95% CI = −0.89, 1.84; *p* = 0.49; n = 4; Figure 7). However, subgroup analysis showed that the IL-2 level was lower in patients taking GCs than HCs (SMD = −0.53; 95% CI = −0.83, −0.23; *p* = 0.0005; n = 2; Figure 7), but higher in patients not using GCs than HCs (SMD = 1.63; 95% CI = 0.07, 3.19; *p* = 0.04; n = 2; Figure 7). Notably, patients in the Diana et al. study (Salazar-Camarena et al., 2019) did not use any medications, and those in the Pablo Medrano-Campillo et al. study (Medrano-Campillo et al., 2015) received hydroxychloroquine, diclofenac, ibuprofen, or other medications, and the heterogeneity of these studies could not be eliminated.

#### Th2 cytokines

**FIGURE 8:**
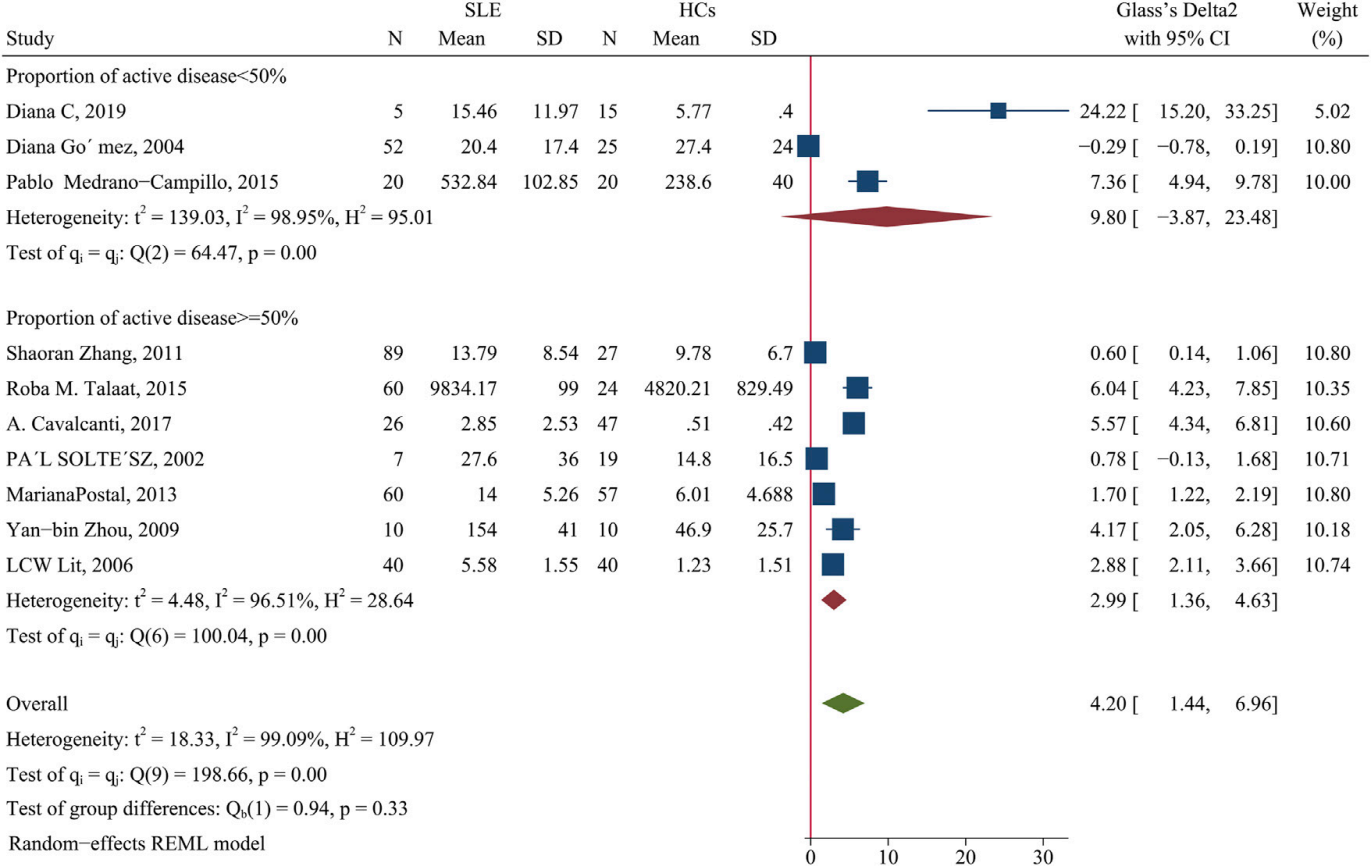
Forest plot of the concentration change of IL-10 in SLE patients compared with HCs. The overall results (bottom) show the meta-analysis of 10 studies, and the other two results show meta-analyses of subgroups according to the percentage of patients with active disease. SLE, systemic lupus erythematosus; HCs, healthy controls.

Patients had a higher level of IL-10 than HCs (SMD = 4.2; 95% CI = 1.44, 6.96; *p* = 0.003; n = 10; Figure 8). Subgroup analysis showed that this difference was present in studies in which more than 50% of the patients had active disease (SMD = 2.99; 95% CI = 1.36, 4.63; *p* < 0.001; n = 7; Figure 8), but not in studies in which fewer than 50% of the patients had active disease.

We performed another subgroup analysis to compare studies that had more than 94% women with those that had 94% or fewer women, with exclusion of the Diana C et al. study (Salazar-Camarena et al., 2019) because it did not provide the requisite data. The results showed that when the proportion of female patients was more than 94%, the IL-10 level was not significantly different in patients and HCs; however, this difference was significant when the proportion of females was less than 94% (SMD = 3.34; 95% CI = 1.01, 5.67; *p* = 0.005; Table 2).

**TABLE 2:** Subgroup analysis in IL-10 according to the proportion of female SLE patients in total patients and the use of medication.

	No. of Studies	SMD	*p*-Value (%)	I^2^ (%)	*p*-Value for Heterogeneity	95% CI
Proportion of women						
Proportion<94	5	3.34	0.005	95.88	<0.001	1.01 to 5.67
Proportion ≥94%	4	2.75	0.073	98.70	<0.001	−0.25 to 5.76
Medication use						
No	2	2.27	0.001	54.90	0.137	0.95 to 3.60
Yes	8	2.45	<0.001	96.80	<0.001	1.24 to 3.66

SMD, standardized mean differences.

**FIGURE 9:**
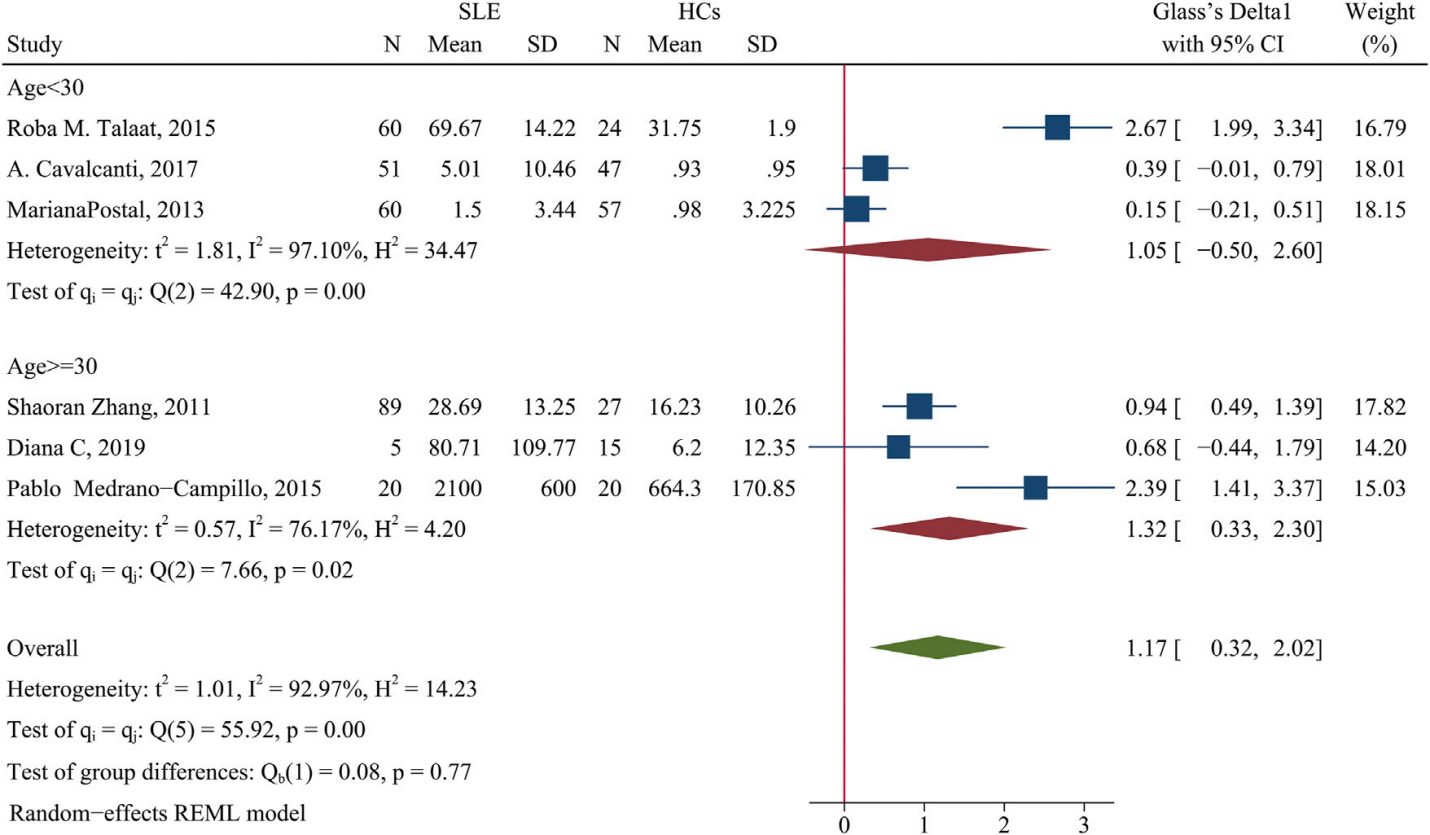
Forest plot of the concentration change of IL-6 in SLE patients compared with HCs. The overall results (bottom) show the meta-analysis of 6 studies, and the other two results show meta-analyses of subgroups according to patient age. SLE, systemic lupus erythematosus; HCs, healthy controls.

The level of IL-6 was higher in patients than HCs (SMD = 1.17; 95% CI = 0.32, 2.02; *p* = 0.007; n = 6; Figure 9). A subgroup analysis showed that when the average patient age was 30 years-old or more, the concentration of IL-6 was higher in patients than HCs (SMD = 1.32; 95% CI = 0.33, 2.30; *p* = 0.009; n = 3; Figure 9), but there was no significant difference when the average patient age was less than 30 years-old (*p* > 0.05).

Sensitivity analysis of the effect of age indicated the heterogeneity between these two groups was eliminated after excluding Roba M. Talaat study (Talaat et al., 2015) and the Pablo Medrano-Campillo study (Medrano-Campillo et al., 2015), but the results did not change (Supplementary Figure S8). The use of GCs also affected the concentration of IL-6. When more than 90% of patients used GCs, the concentration of IL-6 cells was higher in patients than HCs (SMD = 1.78; 95% CI = 0.09, 3.48; *p* = 0.039; n = 2; Table 3). This suggests that GC use may increase the concentration of IL-6. In addition, the proportion of female patients in a study had no effect on IL-6 concentration (Table 3).

**TABLE 3:** Subgroup analysis in IL-6 according to the proportion of female SLE patients in total patients and the use of glucocorticoid.

	No. of Studies	SMD	*p*-Value (%)	I^2^ (%)	*p*-Value for Heterogeneity	95% CI
Proportion of women						
Proportion ≥94%	2	1.22	0.275	94.33	<0.001	−0.97 to 3.42
Proportion<94%	3	1.13	0.053	95.34	<0.001	−0.02 to 2.63
Percentage of taking glucocorticoids						
Percentage≥90%	2	1.78	0.039	94.23	0.024	0.09 to 3.48
Percentage≥50%∼90%	2	0.26	0.061	0	0.389	−0.01 to 0.53
Percentage<50%	2	1.56	0.069	80.43	<0.001	−0.12 to 3.24

SMD, standardized mean differences.

**FIGURE 10:**
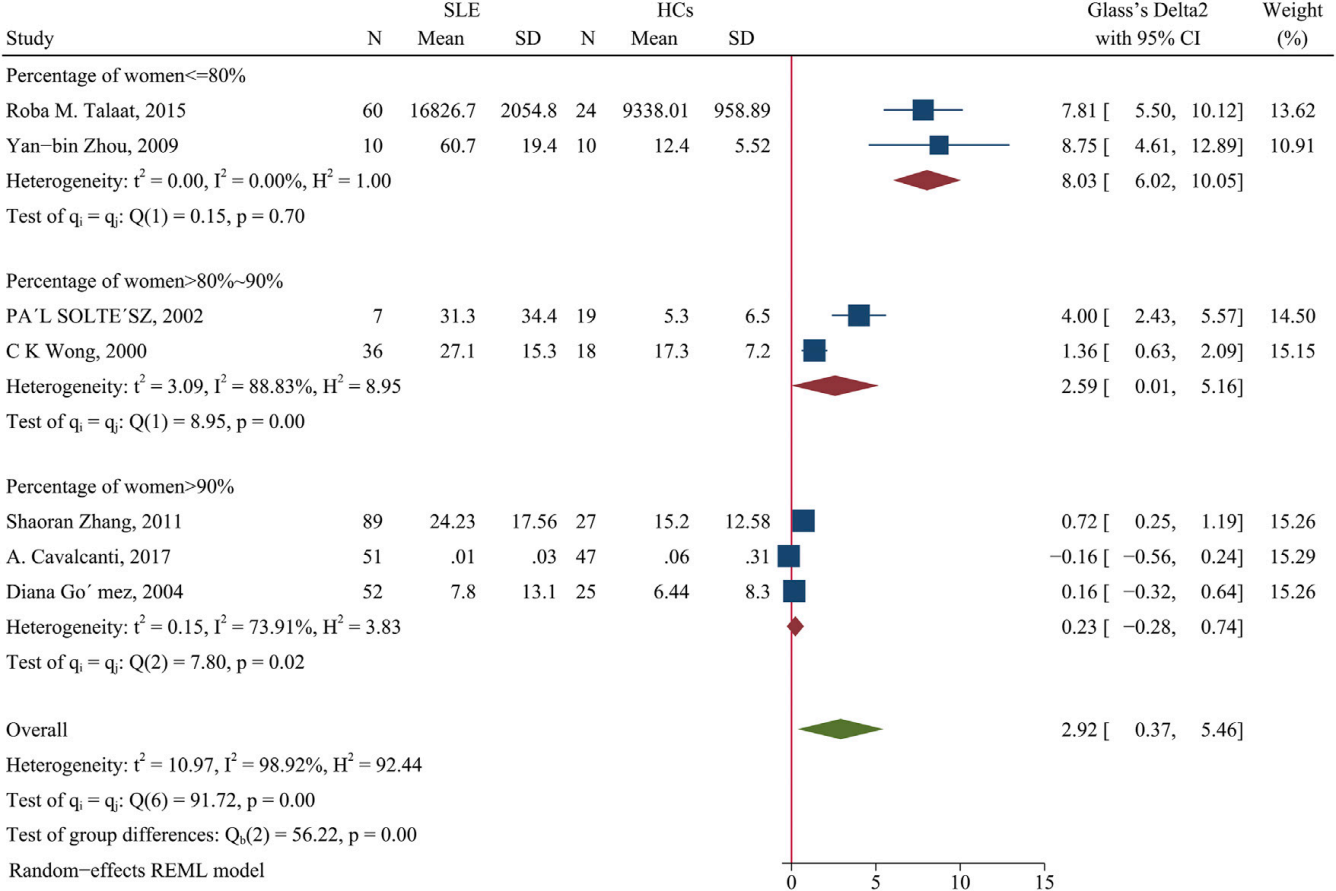
Forest plot of the concentration change of IL-4 in SLE patients compared with HCs. The overall results (bottom) show the meta-analysis of 7 studies, and the other three results show meta-analyses of subgroups according to the percentage of females. SLE, systemic lupus erythematosus; HCs, healthy controls.

Patients had a higher IL-4 concentration than HCs (SMD = 2.92; 95% CI = 0.37, 5.46; *p* = 0.02; n = 7; Figure 10). We performed a subgroup analysis of studies in which the proportion of females was 90% or more; more than 80% and less than 90%; and 80% or less. The results showed no difference of patients and HCs in studies that had 90% or more females (*p* > 0.05). However, when the proportion of women 80–90%, the concentration of IL-4 was higher in patients than HCs (SMD = 2.59; 95% CI = 0.01, 5.16; *p* = 0.049; Figure 10) and when the proportion of females was 80% or less, the concentration of IL-4 was even greater in patients than HCs (SMD = 8.03; 95% CI = 6.02, 10.05; *p* < 0. 001; Figure 10). In addition, a subgroup analysis showed no significant difference in the IL-4 level between patients using a GC and HCs (*p* > 0.05; Table 4).

**TABLE 4:** Subgroup analysis in IL-4 according to the use of glucocorticoid in SLE patients.

	No. of Studies	SMD	*p*-Value (%)	I^2^ (%)	*p*-Value for Heterogeneity	95% CI
Taking glucocorticoids						
Yes	5	0.05	0.916	92.24	<0.001	−0.84 to 0.94
No	2	3.23	<0.001	46.36	0.17	2.14 to 4.32

SMD, standardized mean differences.

### Analysis of publication bias

We assessed the risk of publication bias using the Egger test, Begg test, and a funnel plot (Supplementary Figure S9 and Supplementary Table S2). The results showed no evidence of publication bias in most studies, suggesting that the conclusions of this meta-analysis were relatively robust. However, there was some evidence of publication bias regarding the results for IL-10 and IL-4.

## Discussion

The results of the present meta-analysis showed that SLE patients had significantly lower percentage of Th1 cells, a higher percentage of Th2 cells and higher levels of Th1- and Th2-associated cytokines (IFN-γ, TNF-α, IL-2, IL-10, IL-6 and IL-4) than HCs. Moreover, disease activity appeared unrelated to the proportion of Th1 and Th2 cells.

SLE is characterized by polyclonal activation of B cells, which leads to the production of autoantibodies and pathological damage. Several cytokines secreted by Th2 cells promote the production of B cell antibodies (O'Brien et al., 2021). There is evidence that a negative feedback between Th1-type and Th2-type immune responses regulates normal immune balance. In SLE patients, an influx of Th2 cells inhibits the release of Th1 cells, thus supporting the hypothesis that the immune disorder of “Th2 dominance” is responsible for SLE (Ko et al., 2022).

However, we found a significantly higher proportion of Th1 cells in patients with abnormal kidney function. Previous studies showed that up-regulation of lipocalin-2 in SLE can promote the *in vivo* and *in vitro* differentiation of Th1 cells via the IL-12/STAT4 signalling pathway, thereby causing an immune imbalance and leading to lupus nephritis (LN) (Chen et al., 2020). Our study also showed that the level of Th2 cells was not greater in patients with abnormal kidney function, similar to previous studies (Uhm et al., 2003), and indicating an imbalanced Th1/Th2 ratio in LN.

Studies have shown that medications have significant effects on the level of Th1, Th2 and their associated cytokine levels in patients with systemic lupus erythematosus.

The different results regarding Th1 cells in previous studies of SLE patients may be attributed to whether or not these patients were receiving treatment, especially by GCs. Previous studies showed that GCs have a strong inhibitory effect on Th1 cells by suppressing the production and signal transduction mediated by IL-12 and IFN- γ and that GCs had a weaker effect on Th2 cells, and had little effect on IL-4-induced STAT6 phosphorylation (Franchimont et al., 2000; Taves and Ashwell, 2021). However, our meta-analysis results showed that untreated SLE patients had a lower proportion of Th1 cells relative to treated patients. This may be because Th1 cells mainly target exogenous antigens, such as infected and deteriorating cells (Hall, 2015), and cellular immunity mediated by Th1 cells does not play a major role in SLE because it is an antibody-mediated immune disease. We found that the percentage of Th1 cells was not significantly different in medicated patients and HCs. This may be because the medication reduced the autoimmune response of SLE by increasing the percentage of Th1 cells to achieve a relative balance in Th1 and Th2 levels.

Drug therapy can lead to the suppression of Th1-mediated production of IFN-γ. For example, a previous study showed that IFN-γ-responsive genes were significantly upregulated in SLE patients with untreated active disease relative to HCs, but there was no significant difference in a comparison of HCs with SLE patients who had stable disease due to treatment with GCs, hydroxychloroquine, and/or immunosuppressants (Dufour et al., 2018). However, the use of different dose of GC can lead to different results. For example, Talaat et al. (Talaat et al., 2015) studied 60 SLE patients, including 58 who received a GC dose higher than 10 mg per day, and found that only 32 had active disease. The 2019 European Rheumatic Alliance guidelines for the management of SLE recommend prednisone at a dose lower than 7.5 mg/day when SLE is in remission or low activity (Fanouriakis et al., 2019). Therefore, we hypothesize that the lower level of IFN-γ in medicated patients that were reported in some studies may be due to excessive use of GCs.

Moreover, our results showed the level of IL-2 was higher in SLE patients than HCs, and the use of GC was able to reduce the level of this cytokine. There is evidence that low-dose IL-2 therapy can increase the level of Tregs, and because the lack of Tregs may contribute to the pathogenesis of SLE, this may be an effective treatment for SLE (Tahvildari and Dana, 2019). However, even though IL-2 supplementation significantly increases Treg cells in SLE patients, it does not change the total CD4^+^ cells (Robinson and Thomas, 2021). This may be because SLE patients have Tregs that secrete less IL-2 and Th1 cells that secrete more IL-2. Although low-dose recombinant human IL-2 can selectively enhance Tregs (rather than Th1 cells) and significantly reduce disease activity (Tahvildari and Dana, 2019), it is unknown whether this treatment also reduces the secretion of IL-2 by Th1 cells. Our results showed that GC treatment of SLE patients effectively reduced the level of IL-2 secreted by Th1 cells. Thus, when considering the use of low-dose IL-2 treatment for SLE patients, it may be necessary to continue GC treatment.

Another interesting finding of this study is that imbalances in the levels of Th1, Th2 and their associated inflammatory cytokines may be associated with disease characteristics in male SLE patients.

The male SLE patients had a higher level of Th2 cells, IL-10 and IL-4 than female patients. The increased IL-10 level is associated with reduced kidney function and increased risk of cardiovascular disease (Yilmaz et al., 2014)^.^ IL-4 producing cells contribute to kidney pathology in SLE patients, and that animals lacking IL-4 had fewer pathological changes in the kidneys and lymph nodes (Reséndiz‐Mora et al., 2021). This may explain why men with SLE have more severe cardiovascular and kidney disease and are more likely to develop kidney failure (Parikh et al., 2020). However, the secretion of IL-10 is affected by many factors, including gender and disease activity, and this may have contributed to the high heterogeneity among the studies we analyzed.

Changes in the Th1/Th2 ratio corroborated the above results regarding the imbalance between Th1 and Th2 cells in SLE patients and the potential underlying causes. For example, medicated SLE patients had a higher Th1/Th2 ratio than HCs, possibly due to an increase in the proportion of Th1 cells. However, there was no significant difference in the Th1Th2 ratio between untreated patients and HCs. We suggest this result occurred because the sex ratio of a study affected the reported level of Th2 cells. The proportion of women was greater than 94% in the untreated subgroup. Here, we found no significant change in the proportion of Th2 cells, although the portion of Th1 cells decreased. Therefore, a change in Th1 cells may not significantly contribute to the imbalanced Th1/Th2 ratio in SLE patients. Interestingly, GCs appeared to primarily affect changes in the number of Th1 cells, not Th2 cells.

Furthermore, we found the effect of age on Th1 and IL-6 levels, which may be associated with the development of lupus nephropathy. The result showed that SLE patients had a higher level of IL-6 than in HCs, although this difference was mainly in studies in which the average patient age was more than 30 years old. Interestingly, Th1 cells were more abundant in patients with abnormal kidney function when the mean age was less than 30 years old, but this difference was not significant in patients when the mean age was greater than 30 years old. Recent research showed that Th1 cytokines functioned in the development of diffuse proliferative LN (Sigdel et al., 2016; Fava et al., 2020) and that Th2 cytokines functioned in the development of membranous LN (Masutani et al., 2004). However, analysis of the antigenic site of IL-6 in the glomerular mesangial cells of patients with LN (Malide et al., 1995) indicated that IL-6 may be closely related to the pathogenesis of membranous LN. Our subgroup analysis indicated that young patients with SLE were less likely to develop end-stage renal disease (ESRD). Another epidemiological investigation found that most children with LN had unchanged or normal kidney function, and that ESRD was rare (Vazzana et al., 2021). In addition, the probability of death from active SLE was much lower in children than adult men (Brunner et al., 2008). These results indicated that the Th1Th/Th2 balance was a critical determinant for the histopathology of LN (Miyake et al., 2011), and the type of LN was related to this balance.

Finally, we also found the contradiction that there was no significant difference in the level of Th1 cells between SLE patients and HCs, even though the patients had a significantly higher level of IFN-γ (Zhong et al., 2017). This may be because patients have increased activation of T cells relative to HCs (Araújo et al., 2016). When the concentration of Th2-associated cytokines was greater than that of Th1-associated cytokines, this may reflect a decreased percentage of Th1 cells and an increased percentage of Th2 cells.

This study has some limitations. All included studies had a retrospective case-control design, indicating the possibility of selection bias and publication bias. In particular, based on the Egger test and Begg test, we identified a statistically significant publication bias in studies that examined the levels of IL-10 and IL-4. Moreover, subgroup analysis cannot completely eliminate the heterogeneity among studies because many factors can affect the secretion of these cytokines. This topic therefore requires further study.

In summary, our results showed that Th2 cells play a crucial role in the pathogenesis of SLE. Notably, we confirmed that SLE patients have abnormal increases of multiple cytokines (IFN-γ, TNF-α, IL-2, IL-10, IL-6, and IL-4) that are associated with Th1 and Th2 cells. Medical therapies, especially GCs, seem to mainly affect Th1 cells. However, excessive use of GCs can apparently lead to an imbalance in the level of Th1 cells. Moreover, there was a higher proportion of Th2 cells in male than female SLE patients, and this abnormality may contribute to the more obvious clinical features, such as higher disease severity and worse long-term prognosis, in male patients. IL-10 and IL-4 may be closely associated with the higher incidence of kidney injury and poor prognosis in males. The higher levels of Th1 cells and IL-6 in different age groups may be associated with the development of LN.

## Data Availability

The original contributions presented in the study are included in the article/Supplementary Material, further inquiries can be directed to the corresponding author.

## Abbreviations

ESRD, End-stage renal disease; GCs, Glucocorticoids; HCs, Healthy controls; INF, Interferon; IL, Interleukin; LN, Lupus nephritis; NOS, Newcastle-Ottawa scale; PROSPERO, Prospective Register of Systematic Reviews; SMDs, Standardized mean differences; SLE, Systemic lupus erythematosus; TNF, Tumor necrosis factor; WMDs, Weighted mean differences.

## References

[B1] Álvarez-Rodríguez L. Martínez-Taboada V. Calvo-Alén J. Beares J. Villa I. López-Hoyos I. (2019). Altered Th17/Treg ratio in peripheral blood of systemic lupus erythematosus but not primary antiphospholipid syndrome. Front. Immunol. 10, 391. 10.3389/fimmu.2019.00391 30894863 PMC6414457

[B2] Amit M. Mor A. Weissgarten J. Rosenberg R. Ramot Y. Wysenbeek A. J. (2000). Inactive systemic lupus erythematosus id associated with a normal stimulated Th(1)/Th(2) cytokine secretory pattern. Cytokine 12, 1405–1408. 10.1006/cyto.2000.0724 10976003

[B3] Aragón C. C. Tafúr R. A. Suárez-Avellaneda A. Martínez M. T. Salas A. L. Tobón G. J. (2020). Urinary biomarkers in lupus nephritis. J. Transl. Autoimmun. 3, 100042. 10.1016/j.jtauto.2020.100042 32743523 PMC7388339

[B4] Araújo J. A. Mesquita D. Jr de Melo Cruvinel W. Salmazi K. I. Kallás E. G. Andrade L. E. (2016). Th17 cells and CD4(+) multifunctional T cells in patients with systemic lupus erythematosus. Rev. Bras. Reumatol. Engl. Ed. 56, 28–36. 10.1016/j.rbre.2015.10.003 27267331

[B5] Bakbergenuly I. Hoaglin D. C. Kulinskaya E. (2020). Estimation in meta-analyses of mean difference and standardized mean difference. Stat. Med. 39, 171–191. 10.1002/sim.8422 31709582 PMC6916299

[B6] Brunner H. I. Gladman D. D. Ibañez D. Urowitz M. D. Silverman E. D. (2008). Difference in disease features between childhood-onset and adult-onset systemic lupus erythematosus. Arthritis Rheum. 58, 556–562. 10.1002/art.23204 18240232

[B7] Catalina M. D. Owen K. A. Labonte A. C. Grammer A. C. Lipsky P. E. (2020). The pathogenesis of systemic lupus erythematosus: Harnessing big data to understand the molecular basis of lupus. J. Autoimmun. 110, 102359. 10.1016/j.jaut.2019.102359 31806421

[B8] Cavalcanti A. Santos R. Mesquita Z. Duarte A. L. Lucena-Silva N. (2017). Cytokine profile in childhood-onset systemic lupus erythematosus: A cross-sectional and longitudinal study. Braz J. Med. Biol. Res. 50, e5738. 10.1590/1414-431x20175738 28380214 PMC5423750

[B9] Charles N. Hardwick D. Daugas E. Illei G. G. Rivera J. (2010). Basophils and the T helper 2 environment can promote the development of lupus nephritis. Nat. Med. 16, 701–707. 10.1038/nm.2159 20512127 PMC2909583

[B10] Chen W. Li W. Zhang Z. Tang X. Wu S. Yao G. (2020). Lipocalin-2 exacerbates lupus nephritis by promoting Th1 cell differentiation. J. Am. Soc. Nephrol. 31, 2263–2277. 10.1681/asn.2019090937 32646856 PMC7609012

[B11] Davis L. S. Hutcheson J. Mohan C. (2011). The role of cytokines in the pathogenesis and treatment of systemic lupus erythematosus. J. Interferon Cytokine Res. 31, 781–789. 10.1089/jir.2011.0047 21787222 PMC3189549

[B12] Dolff S. Bijl M. Huitema M. G. Limburg P. C. Kallenberg C. G. Abdulahad W. H. (2011). Disturbed Th1, Th2, Th17 and T(reg) balance in patients with systemic lupus erythematosus. Clin. Immunol. 141, 197–204. 10.1016/j.clim.2011.08.005 21920821

[B13] Dufour A. Bellac C. L. Eckhard U. Solis N. Klein T. Kappelhoff R. (2018). C-terminal truncation of IFN-γ inhibits proinflammatory macrophage responses and is deficient in autoimmune disease. Nat. Commun. 9, 2416. 10.1038/s41467-018-04717-4 29925830 PMC6010466

[B14] Fanouriakis A. Kostopoulou M. Alunno A. Aringer M. Bajema I. Boletis J. N. (2019). 2019 update of the EULAR recommendations for the management of systemic lupus erythematosus. Ann. Rheum. Dis. 78, 736–745. 10.1136/annrheumdis-2019-215089 30926722

[B15] Fava A. Buyon J. Mohan C. Zhang T. Belmont H. M. Izmirly P. (2020). Integrated urine proteomics and renal single-cell genomics identify an IFN-γ response gradient in lupus nephritis. JCI Insight 5, e138345. 10.1172/jci.insight.138345 PMC7406291 32396533

[B16] Franchimont D. Galon J. Gadina M. Visconti R. Zhou Y. Aringer M. (2000). Inhibition of Th1 immune response by glucocorticoids: Dexamethasone selectively inhibits IL-12-induced stat4 phosphorylation in T lymphocytes. J. Immunol. 164, 1768–1774. 10.4049/jimmunol.164.4.1768 10657623

[B17] Gómez D. Correa P. A. Gómez L. M. Cadena J. Molina J. F. Anaya J. M. (2004). Th1/Th2 cytokines in patients with systemic lupus erythematosus: Is tumor necrosis factor α protective? Seminars Arthritis Rheumatism 33, 404–413. 10.1016/j.semarthrit.2003.11.002 15190525

[B18] Hall B. M. (2015). T cells: Soldiers and spies--the surveillance and control of effector T cells by regulatory T cells. Clin. J. Am. Soc. Nephrol. 10, 2050–2064. 10.2215/cjn.06620714 25876770 PMC4633791

[B19] Han F. Qi W. Tang Z. Wang Y. (2004). Correlation between systemic lupus erythematosus and peripheral blood T cell subtypes. Chin Med Clin. 5, 385–387. 10.3969/j.issn.1671-2560.2004.05.025

[B20] Hayashino Y. Noguchi Y. Fukui T. (2005). Systematic evaluation and comparison of statistical tests for publication bias. J. Epidemiol. 15, 235–243. 10.2188/jea.15.235 16276033 PMC7904376

[B21] Higgins J. P. Thompson S. G. Deeks J. J. Altman D. G. (2003). Measuring inconsistency in meta-analyses. Bmj 327, 557–560. 10.1136/bmj.327.7414.557 12958120 PMC192859

[B22] Idborg H. Oke V. (2021). Cytokines as biomarkers in systemic lupus erythematosus: Value for diagnosis and drug therapy. Int. J. Mol. Sci. 22, 11327. 10.3390/ijms222111327 34768756 PMC8582965

[B23] Jiang D. Wang L. Bai C. Chen O. (2019). Association between abdominal obesity and asthma: A meta-analysis. Allergy Asthma Clin. Immunol. 15, 16. 10.1186/s13223-019-0333-6 30949213 PMC6431003

[B24] Katsuyama T. Tsokos G. C. Moulton V. R. (2018). Aberrant T cell signaling and subsets in systemic lupus erythematosus. Front. Immunol. 9, 1088. 10.3389/fimmu.2018.01088 29868033 PMC5967272

[B25] Kleczynska W. Jakiela B. Plutecka H. Milewski M. Sanak M. Musial J. (2011). Imbalance between Th17 and regulatory T-cells in systemic lupus erythematosus. Folia Histochem Cytobiol. 49, 646–653. 10.5603/fhc.2011.0088 22252759

[B26] Ko H. Kim C. J. Im S. H. (2022). T Helper 2-associated immunity in the pathogenesis of systemic lupus erythematosus. Front. Immunol. 13, 866549. 10.3389/fimmu.2022.866549 35444658 PMC9014558

[B27] La Cava A. (2010). Anticytokine therapies in systemic lupus erythematosus. Immunotherapy 2, 575–582. 10.2217/imt.10.29 20636010 PMC2929574

[B28] Li L. Chen S. Shen N. Wang X. Bao C. Gu Y. (2002). Study on Th1/Th2 and its regulatory factor IL-10, IL-12 gene in patients with primary systemic lupus erythematosus. Chin J Rheumatol 1, 13–17. 10.3760/j:issn:1007-7480.2002.01.003

[B29] Li L. Wang X. Chen S. Shen N. Bao C. Gu Y. (2002). Expression of Th1/Th2, IL-10 and IL-18 genes in patients with first-onset SLE. Shanghai J Immunol 03, 163–167. 10.3969/j.issn.1001-2478.2002.03.005

[B30] Liao X. Li G. Wang A. Liu T. Feng S. Guo Z. (2015). Repetitive transcranial magnetic stimulation as an alternative therapy for cognitive impairment in alzheimer's disease: A meta-analysis. J. Alzheimers Dis. 48, 463–472. 10.3233/jad-150346 26402010

[B31] Lit L. C. Wong C. K. Tam L. S. Li E. K. Lam C. W. (2006). Raised plasma concentration and *ex vivo* production of inflammatory chemokines in patients with systemic lupus erythematosus. Ann. Rheum. Dis. 65, 209–215. 10.1136/ard.2005.038315 15975968 PMC1798029

[B32] Liu X. Qian J. (2005). Study on the balance of Th1/Th2 cells in peripheral blood of patients with systemic lupus erythematosus. J Nant. Univ. (Med Ed. 4, 253–254. 10.3969/j.issn.1674-7887.2005.04.007

[B33] Lourenco E. V. Cava A. (2009). Cytokines in systemic lupus erythematosus. Cmm 9, 242–254. 10.2174/156652409787847263 PMC3589140 19355907

[B34] Malide D. Russo P. Bendayan M. (1995). Presence of tumor necrosis factor alpha and interleukin-6 in renal mesangial cells of lupus nephritis patients. Hum. Pathol. 26, 558–564. 10.1016/0046-8177(95)90253-8 7750940

[B35] Masutani K. Taniguchi M. Nakashima H. Yotsueda H. Kudoh Y. Tsuruya K. (2004). Up-regulated interleukin-4 production by peripheral T-helper cells in idiopathic membranous nephropathy. Nephrol. Dial. Transpl. 19, 580–586. 10.1093/ndt/gfg572 14767012

[B36] Matia-Garcia I. Vadillo E. Pelayo R. Muñoz-Valle J. F. García-Chagollán M. Loaeza-Loaeza J. (2021). Th1/Th2 balance in young subjects: Relationship with cytokine levels and metabolic profile. J. Inflamm. Res. 14, 6587–6600. 10.2147/JIR.S342545 34908860 PMC8664383

[B37] Medrano-Campillo P. Sarmiento-Soto H. Álvarez-Sánchez N. Álvarez-Ríos A. I. Guerrero J. M. Rodríguez-Prieto I. (2015). Evaluation of the immunomodulatory effect of melatonin on the T-cell response in peripheral blood from systemic lupus erythematosus patients. J. Pineal Res. 58, 219–226. 10.1111/jpi.12208 25612066

[B38] Miyake K. Akahoshi M. Nakashima H. (2011). Th subset balance in lupus nephritis. J. Biomed. Biotechnol. 2011, 980286. 10.1155/2011/980286 21904445 PMC3163408

[B39] Moulton V. R. Holcomb D. R. Zajdel M. C. Tsokos G. C. (2012). Estrogen upregulates cyclic AMP response element modulator α expression and downregulates interleukin-2 production by human T lymphocytes. Mol. Med. 18, 370–378. 10.2119/molmed.2011.00506 22281835 PMC3356426

[B40] Moulton V. R. Tsokos G. C. (2011). Abnormalities of T cell signaling in systemic lupus erythematosus. Arthritis Res. Ther. 13, 207. 10.1186/ar3251 21457530 PMC3132009

[B41] Muhammad Yusoff F. Wong K. K. Mohd Redzwan N. (2020). Th1, Th2, and Th17 cytokines in systemic lupus erythematosus. Autoimmunity 53, 8–20. 10.1080/08916934.2019.1693545 31771364

[B42] Murphy K. A. Bhamidipati K. Rubin S. J. S. Kipp L. Robinson W. H. Lanz T. V. (2019). Immunomodulatory receptors are differentially expressed in B and T cell subsets relevant to autoimmune disease. Clin. Immunol. 209, 108276. 10.1016/j.clim.2019.108276 31669582

[B43] O'Brien S. A. Zhu M. Zhang W. (2021). Spontaneous differentiation of T follicular helper cells in LATY136F mutant mice. Front. Immunol. 12, 656817. 10.3389/fimmu.2021.656817 33912184 PMC8072119

[B44] Oster C. Wilde B. Specker C. Sun M. Kribben A. Witzke O. (2019). BTLA expression on Th1, Th2 and Th17 effector T-cells of patients with systemic lupus erythematosus is associated with active disease. Int. J. Mol. Sci. 20, 4505. 10.3390/ijms20184505 PMC6770819 31514450

[B45] Parikh S. V. Almaani S. Brodsky S. Rovin B. H. (2020). Update on lupus nephritis: Core curriculum 2020. Am. J. Kidney Dis. 76, 265–281. 10.1053/j.ajkd.2019.10.017 32220510

[B46] Perry D. J. Titov A. A. Sobel E. S. Brusko L. Morel L. (2020). Immunophenotyping reveals distinct subgroups of lupus patients based on their activated T cell subsets. Clin. Immunol. 221, 108602. 10.1016/j.clim.2020.108602 33007439 PMC8173542

[B47] Postal M. Peliçari K. O. Sinicato N. A. Marini R. Costallat L. T. Appenzeller S. (2013). Th1/Th2 cytokine profile in childhood-onset systemic lupus erythematosus. Cytokine 61, 785–791. 10.1016/j.cyto.2012.11.023 23332615

[B48] Radmanesh F. Mahmoudi M. Yazdanpanah V. Keyvani N. Kia A. R. Nikpoor E. (2020). The immunomodulatory effects of mesenchymal stromal cell-based therapy in human and animal models of systemic lupus erythematosus. IUBMB Life 72, 2366–2381. 10.1002/iub.2387 33006813

[B49] Reséndiz‐Mora A. Wong‐Baeza C. Nevárez‐Lechuga I. Landa‐Saldívar C. Molina‐Gómez E. Hernández‐Pando R. (2021). Interleukin 4 deficiency limits the development of a lupus‐like disease in mice triggered by phospholipids in a non‐bilayer arrangement. Scand. J. Immunol. 93, e13002. 10.1111/sji.13002 33247472

[B50] Robinson S. Thomas R. (2021). Potential for antigen-specific tolerizing immunotherapy in systematic lupus erythematosus. Front. Immunol. 12, 654701. 10.3389/fimmu.2021.654701 34335564 PMC8322693

[B51] Rönnblom L. Leonard D. (2019). Interferon pathway in SLE: One key to unlocking the mystery of the disease. Lupus Sci. Med. 6, e000270. 10.1136/lupus-2018-000270 31497305 PMC6703304

[B52] Salazar-Camarena D. C. Ortíz-Lazareno P. Marín-Rosales M. Cruz A. Muñoz-Valle F. Tapia-Llanos R. (2019). BAFF-R and TACI expression on CD3+ T cells: Interplay among BAFF, APRIL and T helper cytokines profile in systemic lupus erythematosus. Cytokine 114, 115–127. 10.1016/j.cyto.2018.11.008 30467093

[B53] Shen T. Yue Y. He T. Huang C. Qu B. Lv W. (2021). The association between the gut microbiota and Parkinson's disease, a meta-analysis. Front. Aging Neurosci. 13, 636545. 10.3389/fnagi.2021.636545 33643026 PMC7907649

[B54] Sigdel K. R. Duan L. Wang Y. Hu W. Wang N. Sun Q. (2016). Serum cytokines Th1, Th2, and Th17 expression profiling in active lupus nephritis-IV: From a southern Chinese Han population. Mediat. Inflamm. 2016, 4927530. 10.1155/2016/4927530 PMC5055982 27738386

[B55] Stang A. (2010). Critical evaluation of the newcastle-ottawa scale for the assessment of the quality of nonrandomized studies in meta-analyses. Eur. J. Epidemiol. 25, 603–605. 10.1007/s10654-010-9491-z 20652370

[B56] Su D. L. Wang H. J. Ji X. H. Li Y. Y. Xuan H. B. Heng C. (2006). Mycophenolic acid inhibits SLE-associated cytokine expression and promotes apoptosis of peripheral blood mononuclear cells from patients with systemic lupus erythematosus. Acta Pharmacol. Sin. 27, 1051–1057. 10.1111/j.1745-7254.2006.00352.x 16867258

[B57] Tahvildari M. Dana R. (2019). Low-dose IL-2 therapy in transplantation, autoimmunity, and inflammatory diseases. J. Immunol. 203, 2749–2755. 10.4049/jimmunol.1900733 31740549 PMC6986328

[B58] Talaat R. M. Mohamed S. F. Bassyouni I. H. Raouf A. A. (2015). Th1/Th2/Th17/Treg cytokine imbalance in systemic lupus erythematosus (SLE) patients: Correlation with disease activity. Cytokine 72, 146–153. 10.1016/j.cyto.2014.12.027 25647269

[B59] Tang Q. Li G. Liu T. Wang A. Feng S. Liao X. (2015). Modulation of interhemispheric activation balance in motor-related areas of stroke patients with motor recovery: Systematic review and meta-analysis of fMRI studies. Neurosci. Biobehav Rev. 57, 392–400. 10.1016/j.neubiorev.2015.09.003 26344667

[B60] Taves M. D. Ashwell J. D. (2021). Glucocorticoids in T cell development, differentiation and function. Nat. Rev. Immunol. 21, 233–243. 10.1038/s41577-020-00464-0 33149283

[B61] Tsokos G. C. Lo M. S. Costa Reis P. Sullivan K. E. (2016). New insights into the immunopathogenesis of systemic lupus erythematosus. Nat. Rev. Rheumatol. 12, 716–730. 10.1038/nrrheum.2016.186 27872476

[B62] Uhm W. S. Na K. Song G. W. Jung S. S. Lee T. Park M. H. (2003). Cytokine balance in kidney tissue from lupus nephritis patients. Rheumatol. Oxf. 42, 935–938. 10.1093/rheumatology/keg255 12730502

[B63] Vazzana K. M. Daga A. Goilav B. Ogbu E. A. Okamura D. M. Park C. (2021). Principles of pediatric lupus nephritis in a prospective contemporary multi-center cohort. Lupus 30, 1660–1670. 10.1177/09612033211028658 34219529 PMC10461610

[B64] Viallard J. F. Pellegrin J. L. Ranchin V. Schaeverbeke T. Dehais J. Longy-Boursier M. (1999). Th1 (IL-2, interferon-gamma (IFN-gamma)) and Th2 (IL-10, IL-4) cytokine production by peripheral blood mononuclear cells (PBMC) from patients with systemic lupus erythematosus (SLE). Clin. Exp. Immunol. 115, 189–195. 10.1046/j.1365-2249.1999.00766.x 9933441 PMC1905189

[B65] Wahren-Herlenius M. Dörner T. (2013). Immunopathogenic mechanisms of systemic autoimmune disease. Lancet 382, 819–831. 10.1016/s0140-6736(13)60954-x 23993191

[B66] Wan X. Wang W. Liu J. Tong T. (2014). Estimating the sample mean and standard deviation from the sample size, median, range and/or interquartile range. BMC Med. Res. Methodol. 14, 135. 10.1186/1471-2288-14-135 25524443 PMC4383202

[B67] Wang X. Li L. Shen N. Hu D. Gu Y. (2002). The study of Th1/Th2 imbalance in patients with primary systemic lupus erythematosus. Chin J Rheumatol 5, 316–319. 10.3760/j:issn:1007-7480.2002.05.003

[B68] Wang Y. Lian L. Xu J. (2006). Detection of Th0, Th1 and Th2 cell levels in patients with systemic lupus erythematosus. J Anhui Med Univ. 3, 321–323. 10.3969/j.issn.1000-1492.2006.03.028

[B69] Wong C. K. Ho C. Y. Li E. K. Lam C. W. (2020). Elevation of proinflammatory cytokine (IL-18, IL-17, IL-12) and Th2 cytokine (IL-4) concentrations in patients with systemic lupus erythematosus. Lupus 9, 589–593. 10.1191/096120300678828703 11035433

[B70] Wu H. Zhang S. Lin X. He J. Wang S. Zhou P. (2021). Pregnancy-related complications and perinatal outcomes following progesterone supplementation before 20 weeks of pregnancy in spontaneously achieved singleton pregnancies: A systematic review and meta-analysis. Reprod. Biol. Endocrinol. 19, 165. 10.1186/s12958-021-00846-6 34732210 PMC8567546

[B71] Xu W. Li Z. Yang T. Wang B. (2013). Changes and clinical significance of Th17 and Th1 cells and their cytokine levels in patients with systemic lupus erythematosus. Lab. Med. 28, 396–399. 10.3969/j.issn.1673-8640.2013.05.012

[B72] Yang X. Y. Wang H. Y. Zhao X. Y. Wang L. J. Lv Q. H. Wang Q. Q. (2013). Th22, but not th17 might be a good index to predict the tissue involvement of systemic lupus erythematosus. J. Clin. Immunol. 33, 767–774. 10.1007/s10875-013-9878-1 23435610

[B73] Yang Y. (2015). Expression and clinical significance of T helper cell subsets in peripheral blood of systemic lupus erythematosus. Beijing: Chin People's Liberation Army Med College. [dissertation/master’s thesis].

[B74] Yen E. Y. Singh R. R. (2018). Brief report: Lupus-an unrecognized leading cause of death in young females: A population-based study using nationwide death certificates, 2000-2015. Arthritis Rheumatol. 70, 1251–1255. 10.1002/art.40512 29671279 PMC6105528

[B75] Yilmaz M. I. Solak Y. Saglam M. Cayci T. Acikel C. Unal H. U. (2014). The relationship between IL-10 levels and cardiovascular events in patients with CKD. Clin. J. Am. Soc. Nephrol. 9, 1207–1216. 10.2215/CJN.08660813 24789549 PMC4078956

[B76] Zhang S. (2011). Study on the value of combined detection of T helper cell subsets in the evaluation of systemic lupus erythematosus. Shanxi: University of shanxi medicine. [dissertation/doctor’s thesis].

[B77] Zhong W. Jiang Y. Ma H. Wu J. Jiang Z. Zhao L. (2017). Elevated levels of CCR6+ T helper 22 cells correlate with skin and renal impairment in systemic lupus erythematosus. Sci. Rep. 7, 12962. 10.1038/s41598-017-13344-w 29021537 PMC5636893

[B78] Zhou Y. B. Ye R. G. Li Y. J. Xie C. M. (2009). Targeting the CD134-CD134L interaction using anti-CD134 and/or rhCD134 fusion protein as a possible strategy to prevent lupus nephritis. Rheumatol. Int. 29, 417–425. 10.1007/s00296-008-0697-2 18802705

[B79] Zhu J. Yamane H. Paul W. E. (2010). Differentiation of effector CD4 T cell populations (*). Annu. Rev. Immunol. 28, 445–489. 10.1146/annurev-immunol-030409-101212 20192806 PMC3502616

