# Supplementary material for: Imbalance of helper T cell type 1, helper T cell type 2 and associated cytokines in patients with systemic lupus erythematosus: A meta-analysis
Source: Front Pharmacol. 2022 Sep 29;13:988512. doi: 10.3389/fphar.2022.988512 (PMC9556996; doi:10.3389/fphar.2022.988512)
Supplement: Supplementary file 2 [file Table1.docx]

Supplementary Material

# Supplementary Data

**Search strategies for Pubmed, Web of Science, Embase, The Cochrane Library, Wanfang, Chinese National Knowledge Infrastructure databases.**

**Pubmed**

#1.(Lupus Erythematosus, Systemic[mh] OR (Systemic Lupus Erythematosus) OR (Lupus Erythematosus Disseminatus) OR (Libman-Sacks Disease) OR (Disease, Libman-Sacks) OR (Libman Sacks Disease)

#2. (Th1 Cells[mh]) OR (Cell, Th1) OR (Cells, Th1) OR (Th1 Cell) OR (TH-1 Cells) OR (Cell, TH-1) OR (Cells, TH-1) OR (TH 1 Cells) OR (TH-1 Cell) OR (T Helper 1 Cells) OR (Type 1 Helper T Cells) OR (Th2 Cells[mh]) OR (Th2 Cell) OR (Cell, Th2) OR (Cells, Th2) OR (T Helper2 Cell) OR (Cell, T Helper2) OR (Cells, T Helper2) OR (TH-2 Cell) OR (Cell, TH-2) OR (Cells, TH-2) OR (TH 2 Cell) OR (TH-2 Cells) OR (TH 2 Cells) OR (T Helper2 Cells) OR (T Helper 2 Cells) OR (Type-2 Helper T Cells) OR (Type 2 Helper T Cells) OR (T Helper 2 Cell) OR (Type-2 Helper T Cell) OR (Type 2 Helper T Cell)

#3. #2.AND #1.

**Web of Science**

#1. TS= (Lupus Erythematosus, Systemic or Systemic Lupus Erythematosus

or Lupus Erythematosus Disseminatus or Libman-Sacks Disease or Disease, Libman-Sacks or Libman Sacks Disease)

#2. TS= (Th1 Cells or Cell, Th1 or Cells, Th1 or Th1 Cell or TH-1 Cells or Cell, TH-1 or Cells, TH-1 or TH 1 Cells or TH-1 Cell or T Helper 1 Cells or Type 1 Helper T Cells or Th2 Cells or Th2 Cell or Cell, Th2 or Cells, Th2 or T Helper2 Cell or Cell, T Helper2 or Cells, T Helper2 or TH-2 Cell or Cell, TH-2 or Cells, TH-2 or TH 2 Cell or TH-2 Cells or TH 2 Cells or T Helper2 Cells or T Helper 2 Cells or Type-2 Helper T Cells or Type 2 Helper T Cells or T Helper 2 Cell or Type-2 Helper T Cell or Type 2 Helper T Cell)

#3. #2.AND #1.

**Embase**

#1 ‘systemic lupus erythematosus’/exp

#2 ‘lupus erythematosus systemic’: ti, ab, kw OR ‘lupus erythematosus disseminatus’: ti, ab, kw OR ‘libman-sacks disease’: ti, ab, kw OR ‘disease, libman-sacks’: ti, ab, kw OR ‘libman sacks disease’: ti, ab, kw

#3 #1 OR #2

#4 ‘Th1 Cell ’/exp

#5‘Th2 Cell ’/exp

#6 'th1 cells':ti,ab,kw OR 'cell, th1':ti,ab,kw OR 'cells, th1':ti,ab,kw OR 'th1 cell':ti,ab,kw OR 'th-1 cells':ti,ab,kw OR 'cell, th-1':ti,ab,kw OR 'cells, th-1':ti,ab,kw OR 'th 1 cells':ti,ab,kw OR 'th-1 cell':ti,ab,kw OR 't helper 1 cells':ti,ab,kw OR 'type 1 helper t cells':ti,ab,kw OR 't helper1 cell':ti,ab,kw OR 'cell, t helper1':ti,ab,kw OR 'cells, t helper1':ti,ab,kw OR 'th 1 cell':ti,ab,kw OR 't helper1 cells':ti,ab,kw OR 'type-1 helper t cells':ti,ab,kw OR 't helper 1 cell':ti,ab,kw OR 'type-1 helper t cell':ti,ab,kw OR 'type 1 helper t cell':ti,ab,kw

#7 'th2 cells':ti,ab,kw OR 'cell, th2':ti,ab,kw OR 'cells, th2':ti,ab,kw OR 'th2 cell':ti,ab,kw OR 'th-2 cells':ti,ab,kw OR 'cell, th-2':ti,ab,kw OR 'cells, th-2':ti,ab,kw OR 'th 2 cells':ti,ab,kw OR 'th-2 cell':ti,ab,kw OR 't helper 2 cells':ti,ab,kw OR 'type 2 helper t cells':ti,ab,kw OR 't helper2 cell':ti,ab,kw OR 'cell, t helper2':ti,ab,kw OR 'cells, t helper2':ti,ab,kw OR 'th 2 cell':ti,ab,kw OR 't helper2 cells':ti,ab,kw OR 'type-2 helper t cells':ti,ab,kw OR 't helper 2 cell':ti,ab,kw OR 'type-2 helper t cell':ti,ab,kw OR 'type 2 helper t cell':ti,ab,kw

#8 #4OR#5OR#6OR#7

#9 #8AND#3

**Wanfang**

（系统性红斑狼疮 or SLE) AND (辅助型T细胞2 or Th2细胞 or 辅助型T细胞1 or Th1细胞)

**CNKI**

SU=(系统性红斑狼疮+SLE) AND SU=(辅助型T细胞2+Th2细胞+辅助型T细胞1+Th1细胞)

**SinoMed**

#1 "红斑狼疮, 系统性"[不加权:扩展]

#2 "Th1细胞"[不加权:扩展]

#3 "Th2细胞"[不加权:扩展]

#4 (#3) OR (#2)

#5 (#4) AND (#1)

**The cochraen library**

#1MeSH descriptor: [Lupus Erythematosus, Systemic] explode all trees

#2 Libman-Sacks Disease OR Disease, Libman-Sacks OR Libman Sacks Disease OR Lupus Erythematosus Disseminatus OR Systemic Lupus Erythematosus

#3 MeSH descriptor: [Th1 Cells] explode all trees

#4 Cell, Th1 OR TH 1 Cells OR Cells, TH-1 OR T Helper 1 Cells OR Th1 Cell OR Cells, Th1 OR Cell, TH-1 OR TH-1 Cell OR TH-1 Cells OR Type 1 Helper T Cells

#5 MeSH descriptor: [Th2 Cells] explode all trees

#6 Th2 Cell OR Cell, T Helper2 OR T Helper2 Cells OR Cell, Th2 OR Type-2 Helper T Cell OR T Helper 2 Cells OR TH-2 Cells OR Cells, TH-2 OR Cell, TH-2 OR Type-2 Helper T Cells OR TH-2 Cell OR Cells, Th2 OR Cells, T Helper2 OR T Helper2 Cell OR T Helper 2 Cell OR Type 2 Helper T Cells OR TH 2 Cell OR Type 2 Helper T Cell OR TH 2 Cells

#7 #1 OR #2

#8 #3 OR #4

#9 #5 OR #6

#10 #8 OR #9

#11 #10 AND #7

# Supplementary Tables

**Table S1.** Score of studies included in this meta-analysis based on NOS

| Reference | **Selection** | | | |  | **Comparability** |  | **Exposure** | | |  | **Total score** |
| --- | --- | --- | --- | --- | --- | --- | --- | --- | --- | --- | --- | --- |
|  | Adequate Definition of Cases | Representativeness of the Cases | Selection of Controls | Definition of Controls |  | Comparability of Cases and Controls on the Basis of the Design or Analysis |  | Ascertainment of Exposure | Same method of ascertainment for cases and controls | Non-Response Rate |  |  |
| Feng Han  (2004) |  |  |  |  |  |  |  | — |  | — |  | 7 |
| Xiaojuan Liu (2005) |  |  |  |  |  |  |  |  |  | — |  | 8 |
| Weijia Xu  (2013) | 0 |  |  |  |  |  |  |  |  | — |  | 8 |
| Yong Wang (2006) |  |  |  |  |  |  |  |  | — | — |  | 7 |
| Li Li  (2002) |  |  |  |  |  |  |  |  | — | — |  | 7 |
| Shaoran Zhang (2011) |  |  |  |  |  |  |  |  |  | — |  | 8 |
| Xiaodong Wang (2002) |  |  |  |  |  |  |  |  |  | — |  | 7 |
| Xu-yan Yang (2013) |  |  |  |  |  |  |  |  |  | — |  | 8 |
| Yanni Jiang (2021) |  |  |  |  |  |  |  |  |  | — |  | 7 |
| Yufeng Yang (2012) |  |  |  |  |  |  |  |  |  |  |  | 9 |
| Roba M. Talaat (2015) |  |  |  |  |  |  |  |  |  | — |  | 8 |
| Diana C (2019) |  |  |  |  |  |  |  |  |  | — |  | 7 |
| A. Cavalcanti (2017) |  |  |  |  |  |  |  |  |  | — |  | 8 |
| Diana Go´ mez (2004) |  |  |  | — |  |  |  |  |  | — |  | 7 |
| PA´L SOLTE´SZ (2002) |  |  |  |  |  |  |  |  |  | — |  | 8 |
| Pablo Medrano-Campillo (2015) |  |  |  |  |  |  |  |  |  | — |  | 8 |
| Lorena Álvarez-Rodríguez (2019) |  |  |  |  |  |  |  |  |  | — |  | 8 |
| Daniel J Perry (2020) |  |  |  |  |  |  |  |  |  | — |  | 8 |
| Katherine A Murphy (2019) |  |  |  | — |  |  |  |  |  | — |  | 6 |
| Weronika Kleczynska (2011) |  |  |  |  |  |  |  |  |  | — |  | 8 |
| Sebastian Dolff (2011) |  |  |  |  |  |  |  |  |  | — |  | 8 |
| Mariana Postal (2013) |  |  |  |  |  |  |  |  |  |  |  | 9 |
| C K Wong (2000) |  |  |  |  |  |  |  |  |  | — |  | 8 |
|  |  |  |  |  |  |  |  |  |  |  |  |  |
| Ding-lei SU (2006) |  |  |  |  |  |  |  |  |  | — |  | 8 |
| Yan-bin Zhou (2009) |  |  |  |  |  |  |  |  |  | — |  | 8 |
| LCW Lit (2006) |  |  |  |  |  |  |  |  |  | — |  | 8 |

**Table S2.** Egger test and Begg test identifying the publication bias

|  | P > \| t \| (bias) | |
| --- | --- | --- |
|  | **Egger test** | **Begg test** |
| Helper T cell |  |  |
| Th1 cell | 0.263 | 0.263 |
| Th2 cell | 0.509 | 0.509 |
| Disease activity |  |  |
| Th1 cell | 0.244 | 0.244 |
| Th2 cell | 0.094 | 0.094 |
| kidney injury |  |  |
| Th1 cell | 0.319 | 0.319 |
| Th2 cell | 0.908 | 0.908 |
| Cytokines |  |  |
| IFN-γ | 0.606 | 0.606 |
| TNF-α | 0.345 | 0.345 |
| IL-2 | 0.130 | 0.130 |
| IL-10 | 0.005 | 0.005 |
| IL-6 | 0.144 | 0.144 |
| IL-4 | 0.000 | 0.000 |

#
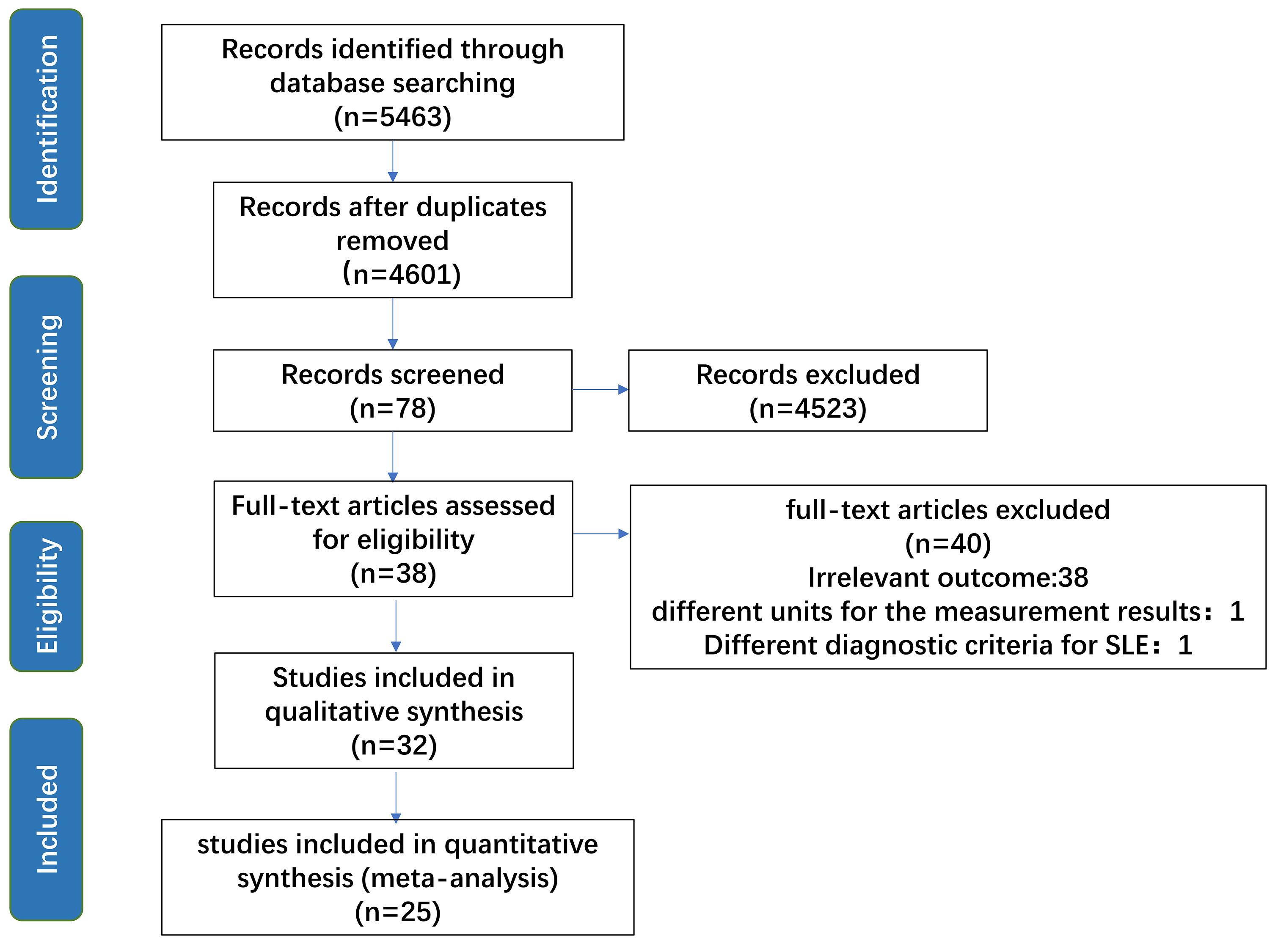
Supplementary Figures

**Supplementary Figure S1.** Flow chart of selection and inclusion of studies in this meta-analysis


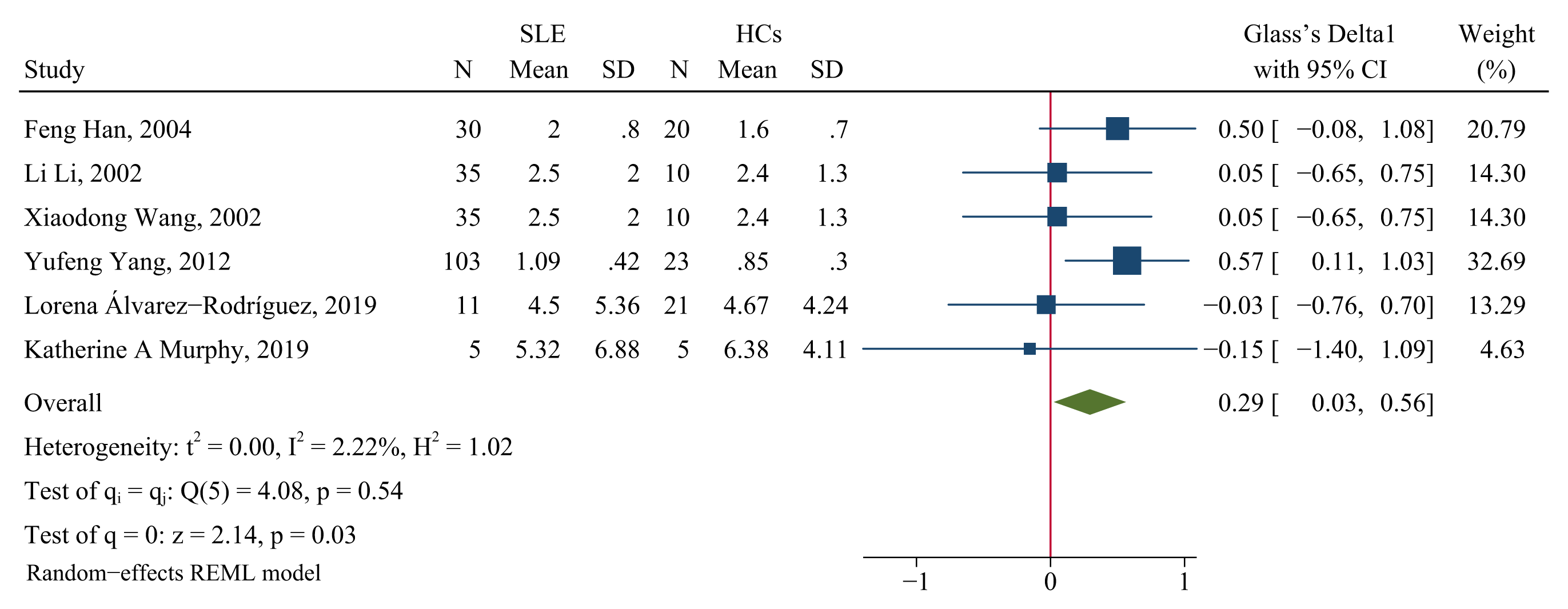


**Supplementary Figure S2.** Forest plot of the percentage changes of Th2 cells in SLE patients compared with the healthy controls after removing Yong Wang (2006) and Yanni Jiang (2021).


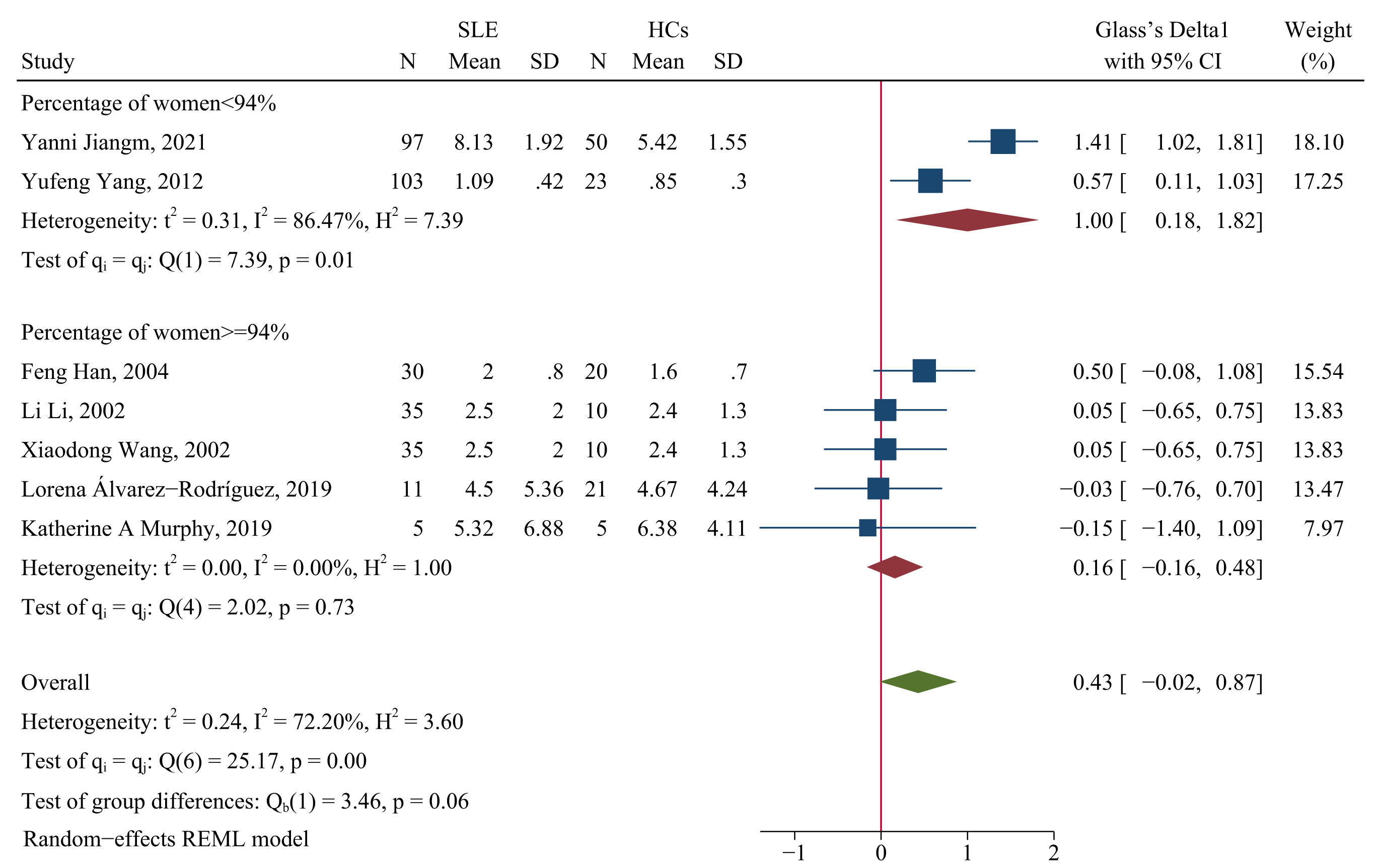


**Supplementary Figure S3.** Subgroup analysis of Th2 cells according to the proportion of female SLE patients in the total patient population (Yong Wang (2006) was removed).


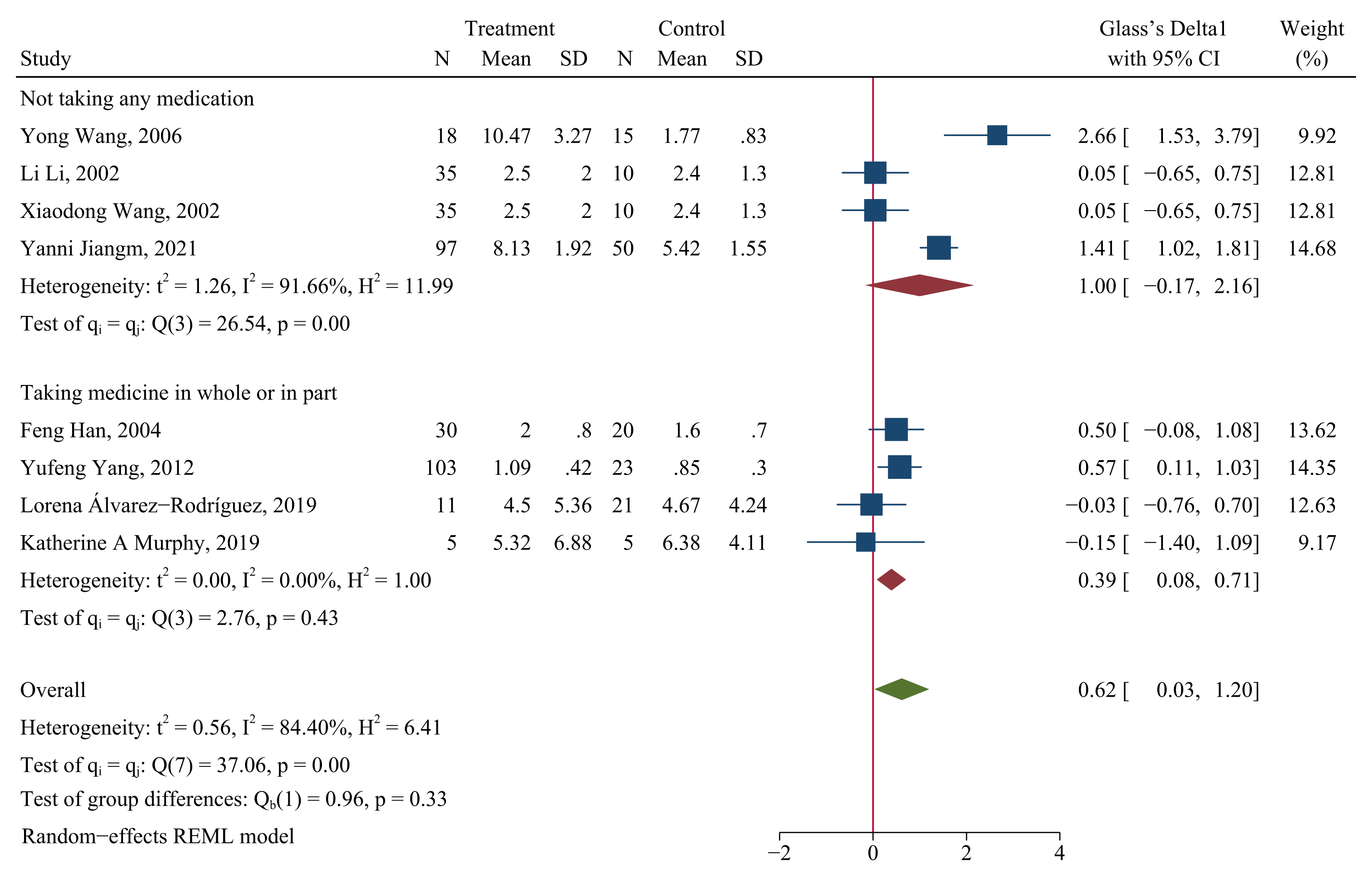


**Supplementary Figure S4.** Subgroup analysis of Th2 cells according to medication use in SLE patients.


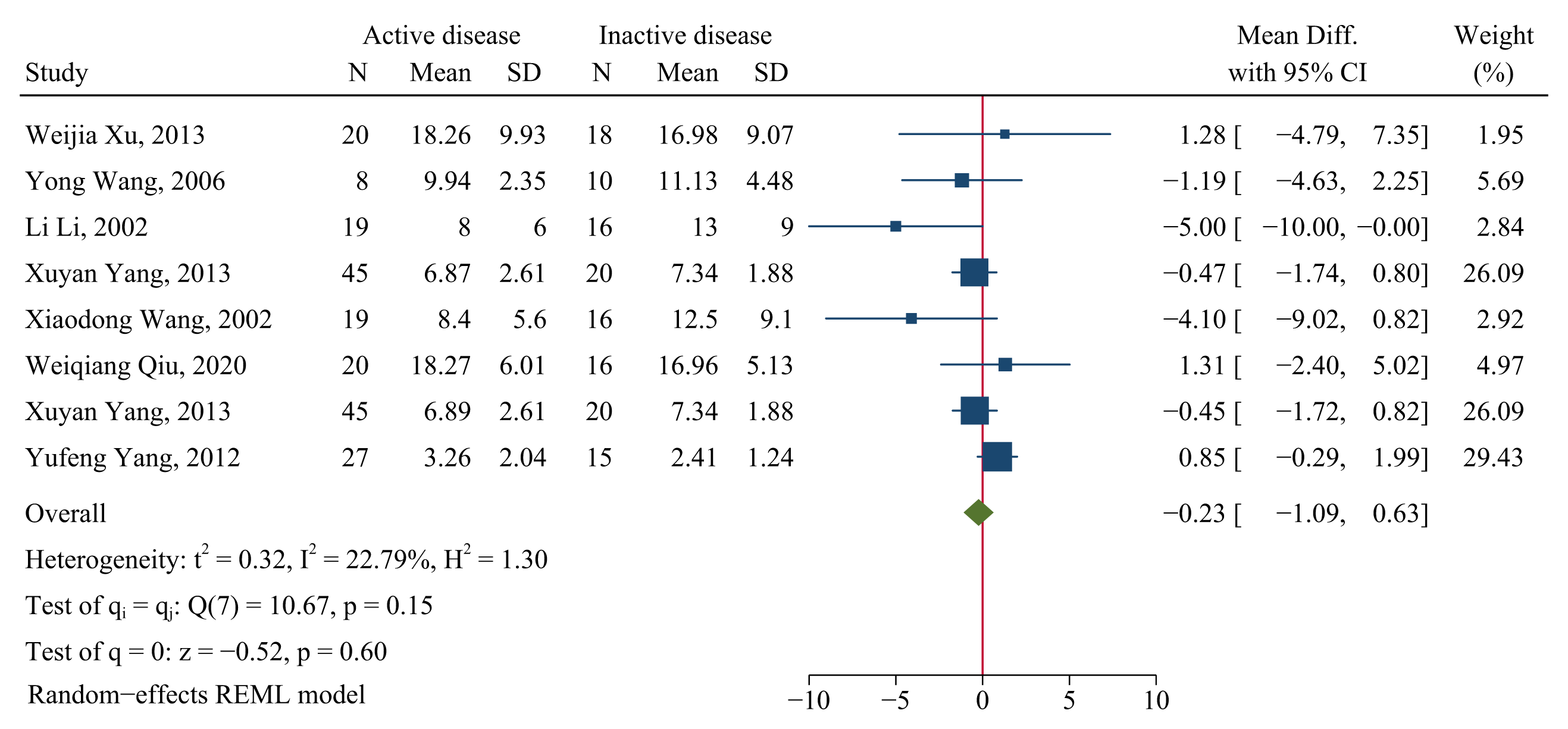


**Supplementary Figure S5.** Forest plots of the percentage changes of Th1 cells in patients with SLE in active disease vs. inactive disease.


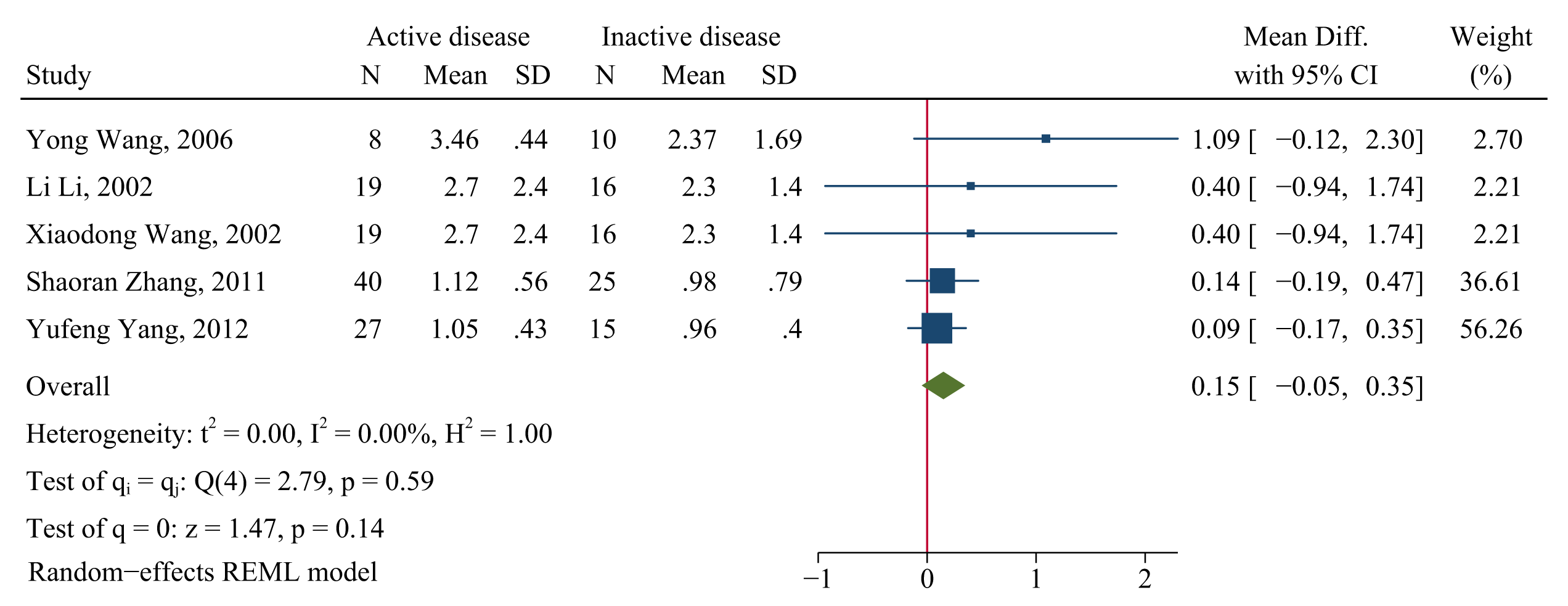


**Supplementary Figure S6.** Forest plots of the percentage changes of Th2 cells in patients with SLE in active disease vs. inactive disease.


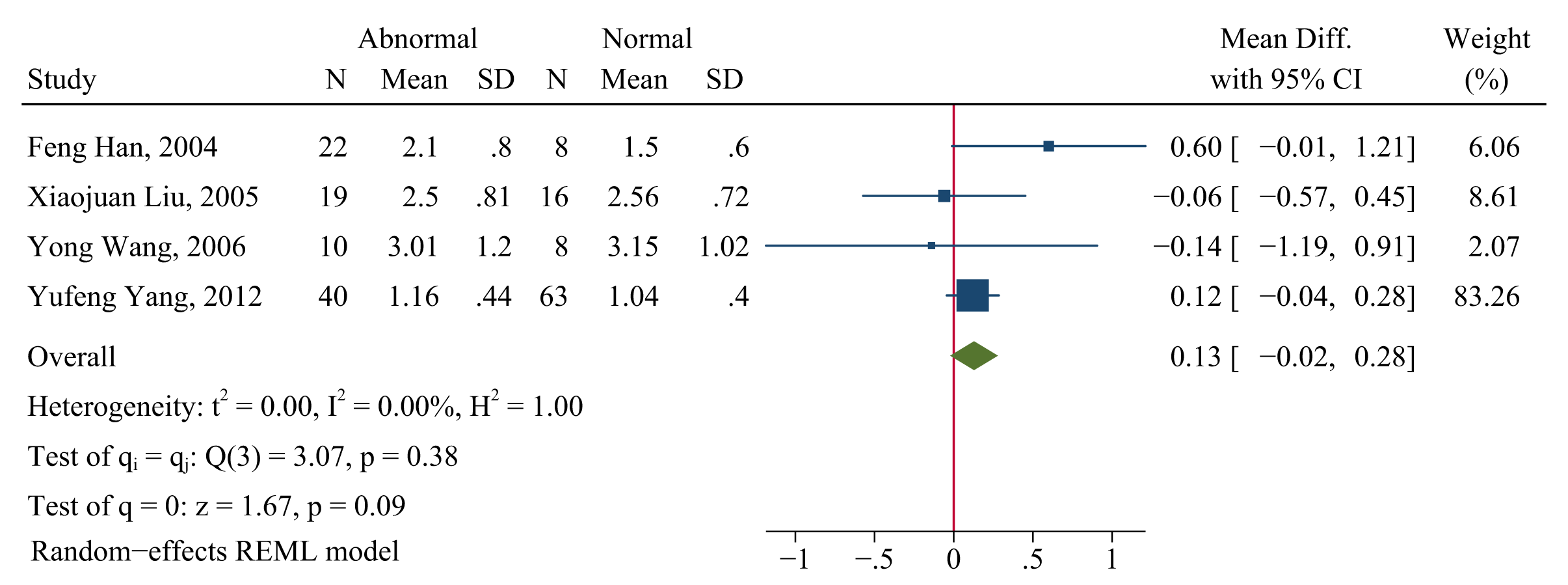


**Supplementary Figure S7.** Subgroup analysis in Th2 cells according to kidney function of SLE patients. Abnormal: SLE patients with abnormal kidney function. Normal: SLE patients with normal kidney function.


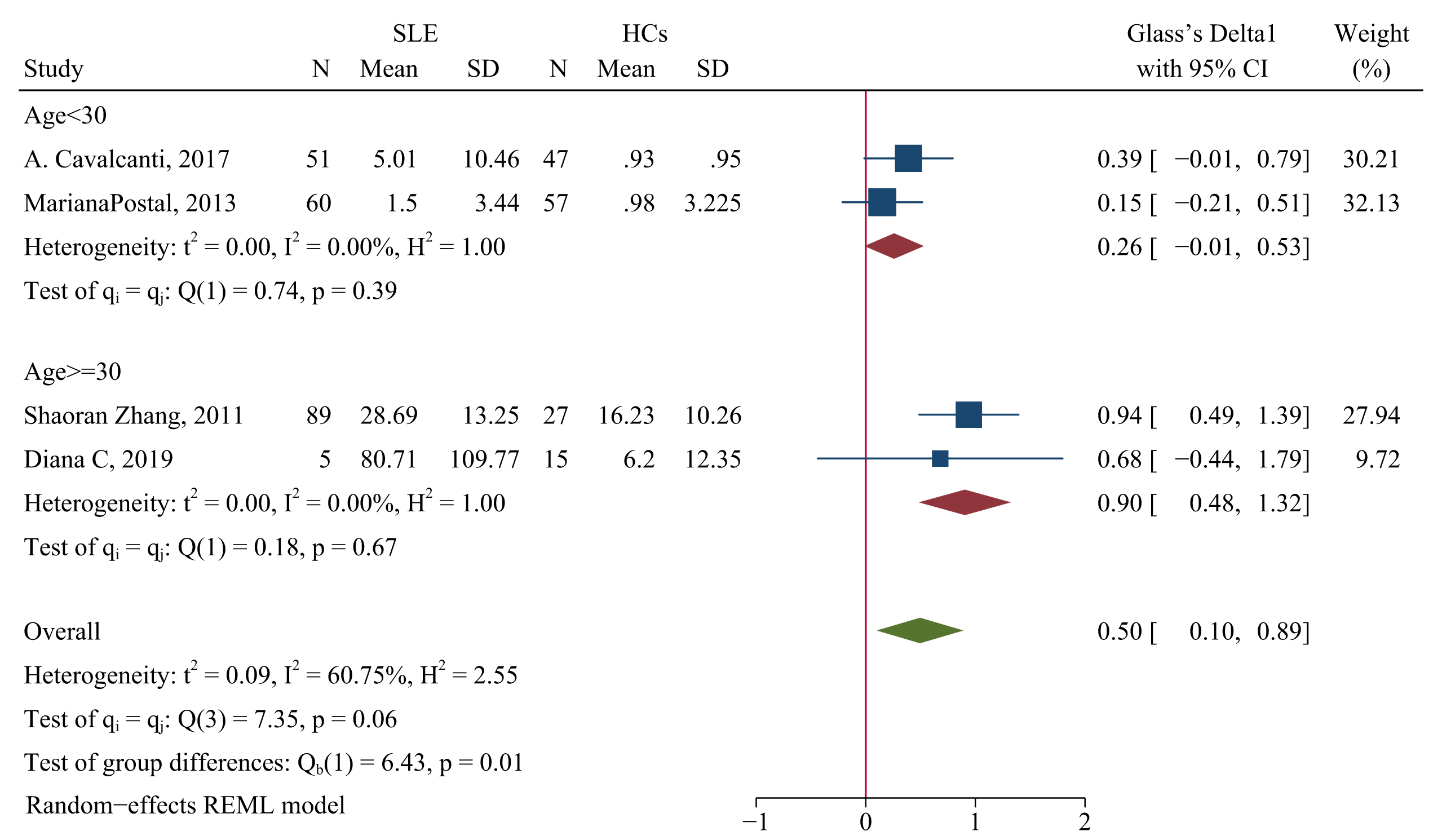


**Supplementary Figure S8.** Subgroup analysis in IL-6 according to the patient age (Roba M. Talaat (2015) and Pablo Medrano-Campillo (2015) was removed). SLE: systemic lupus erythematosus; HCs: healthy controls.


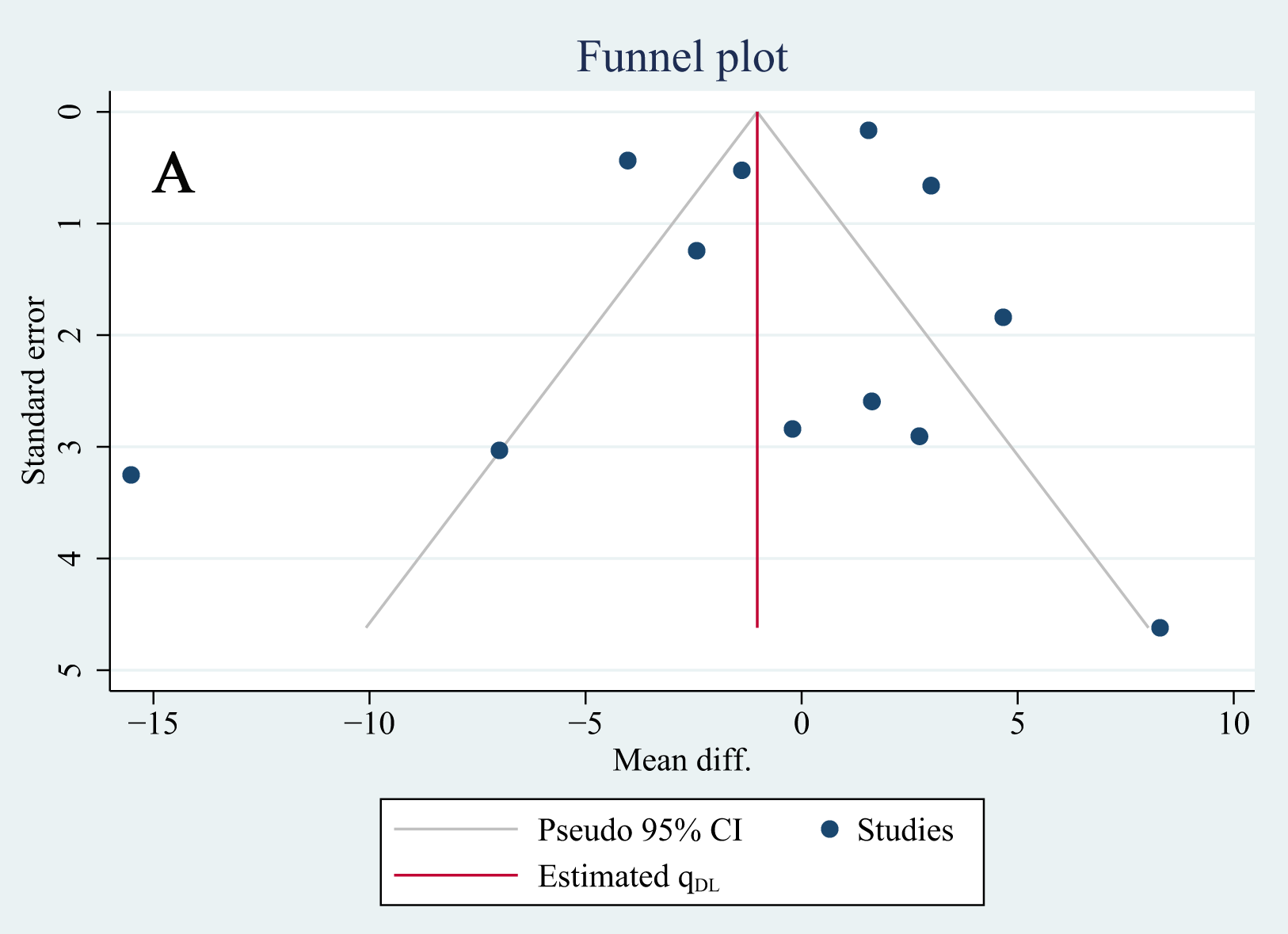


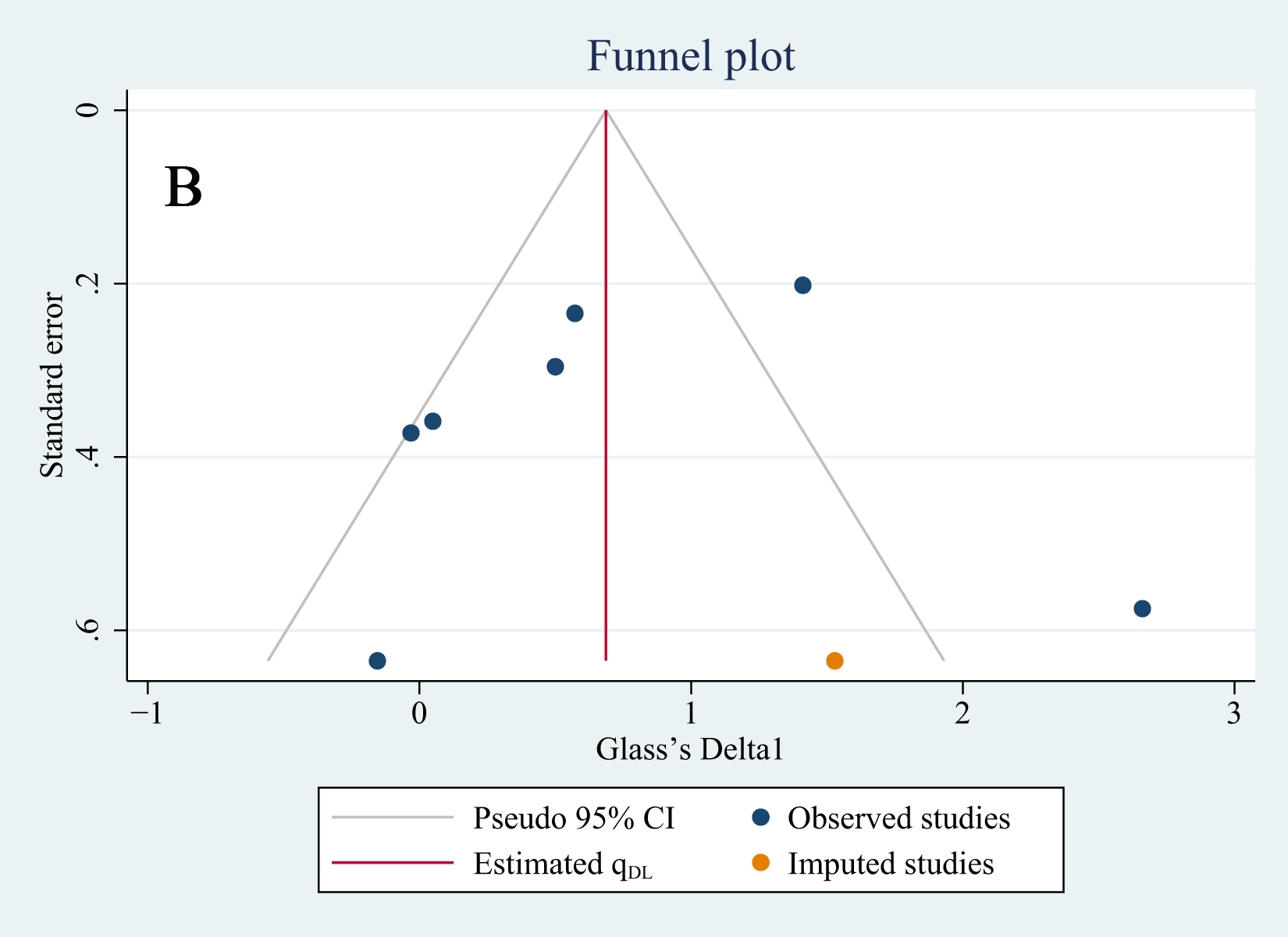


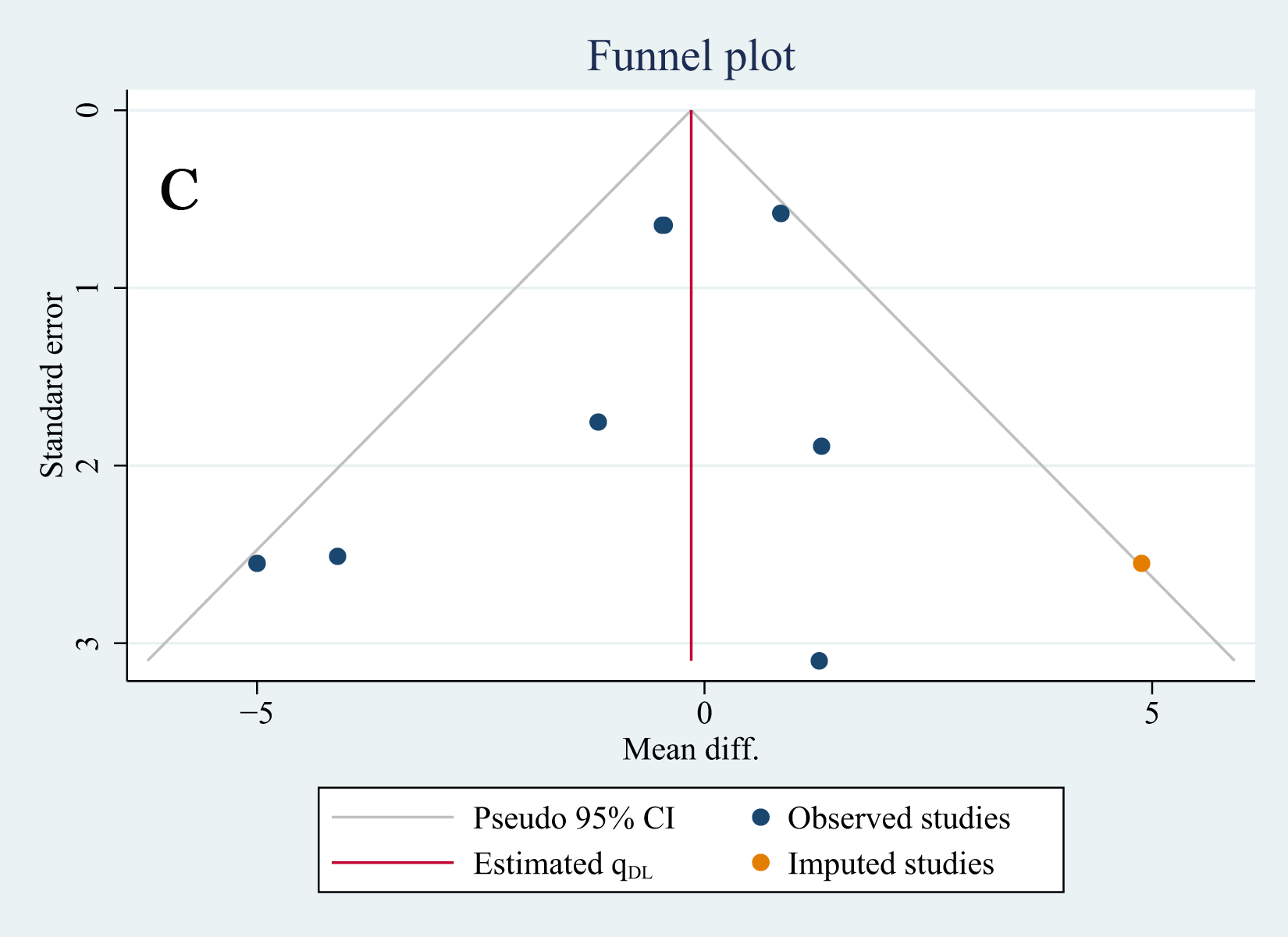


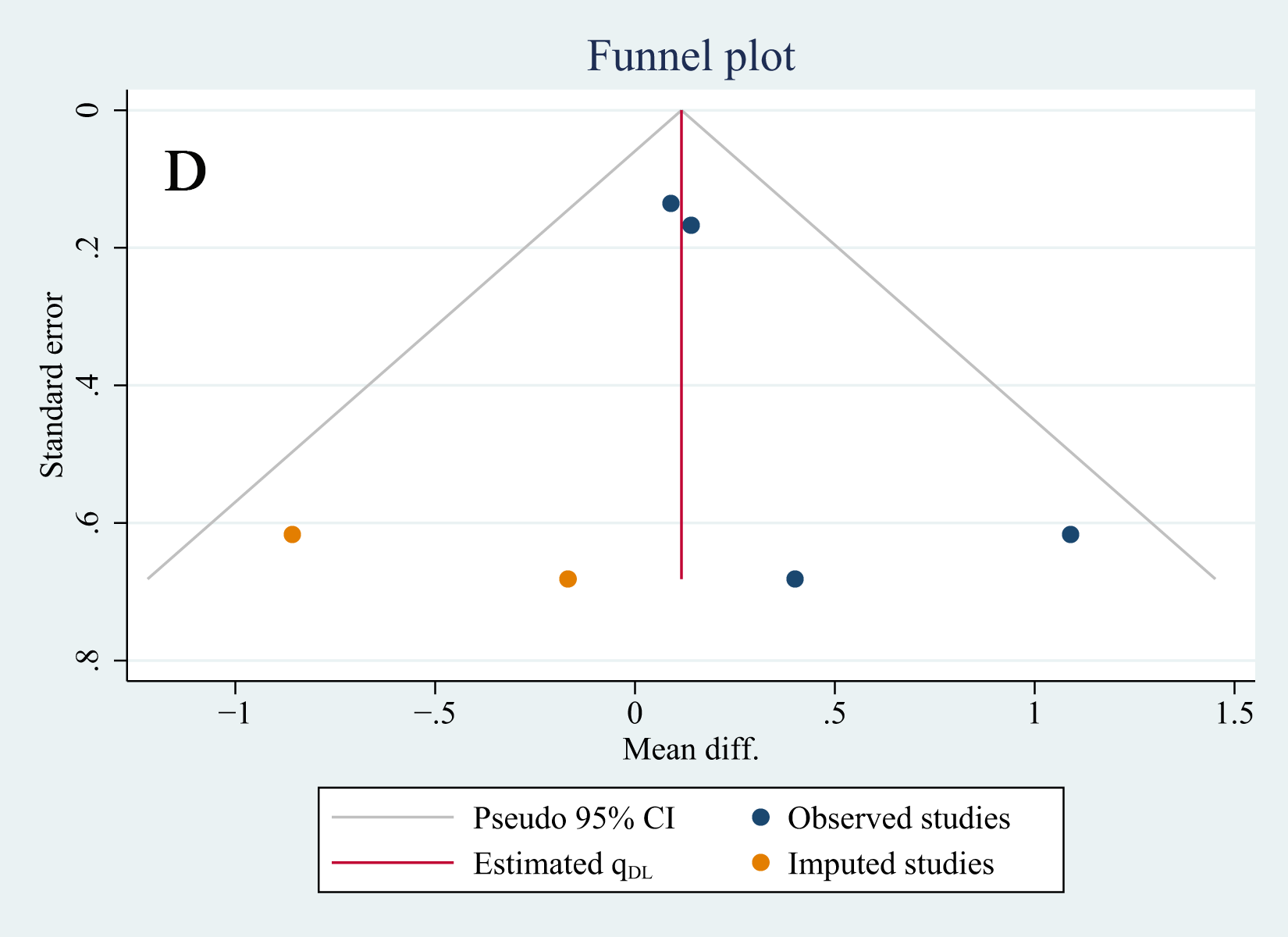


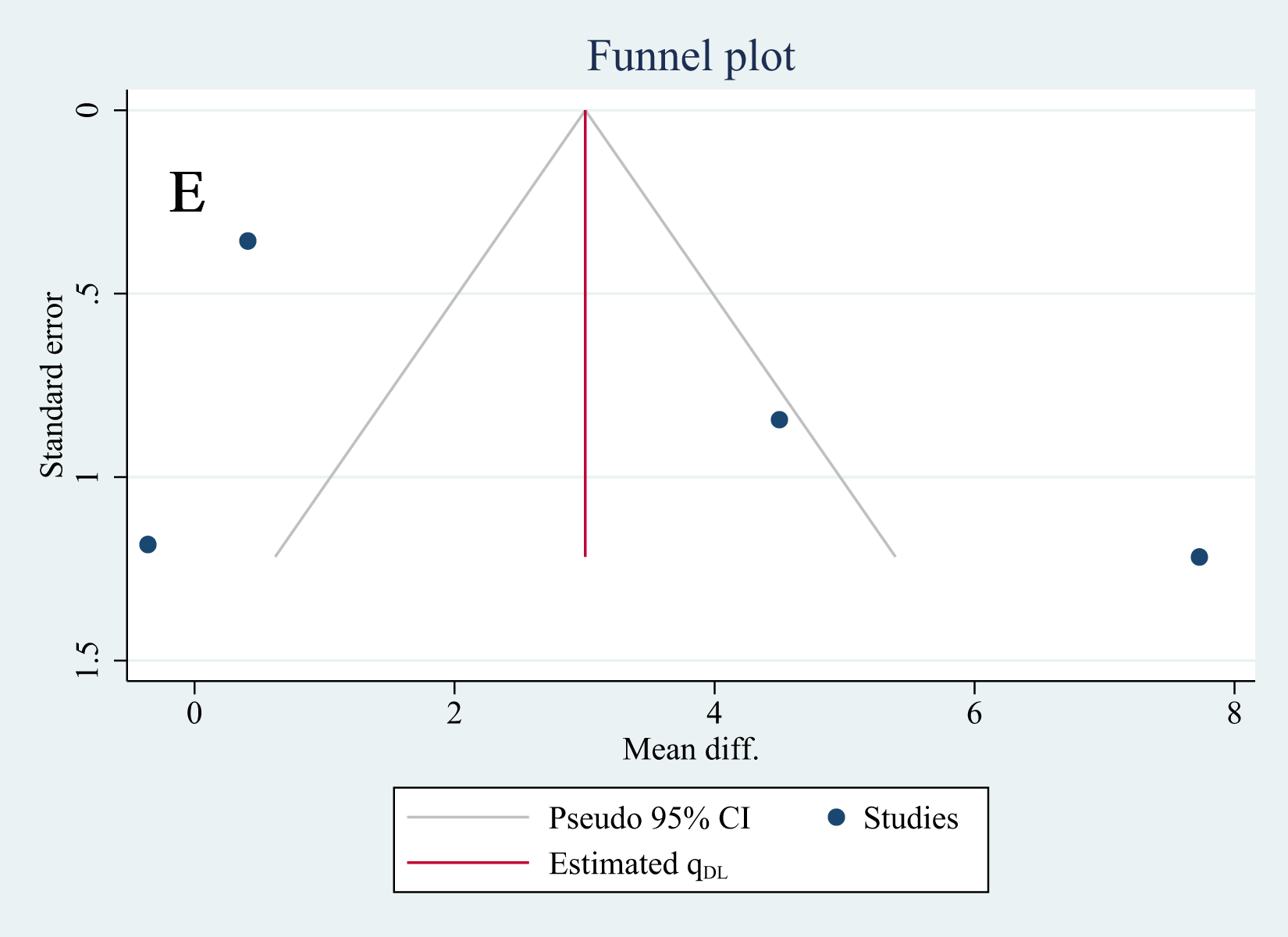


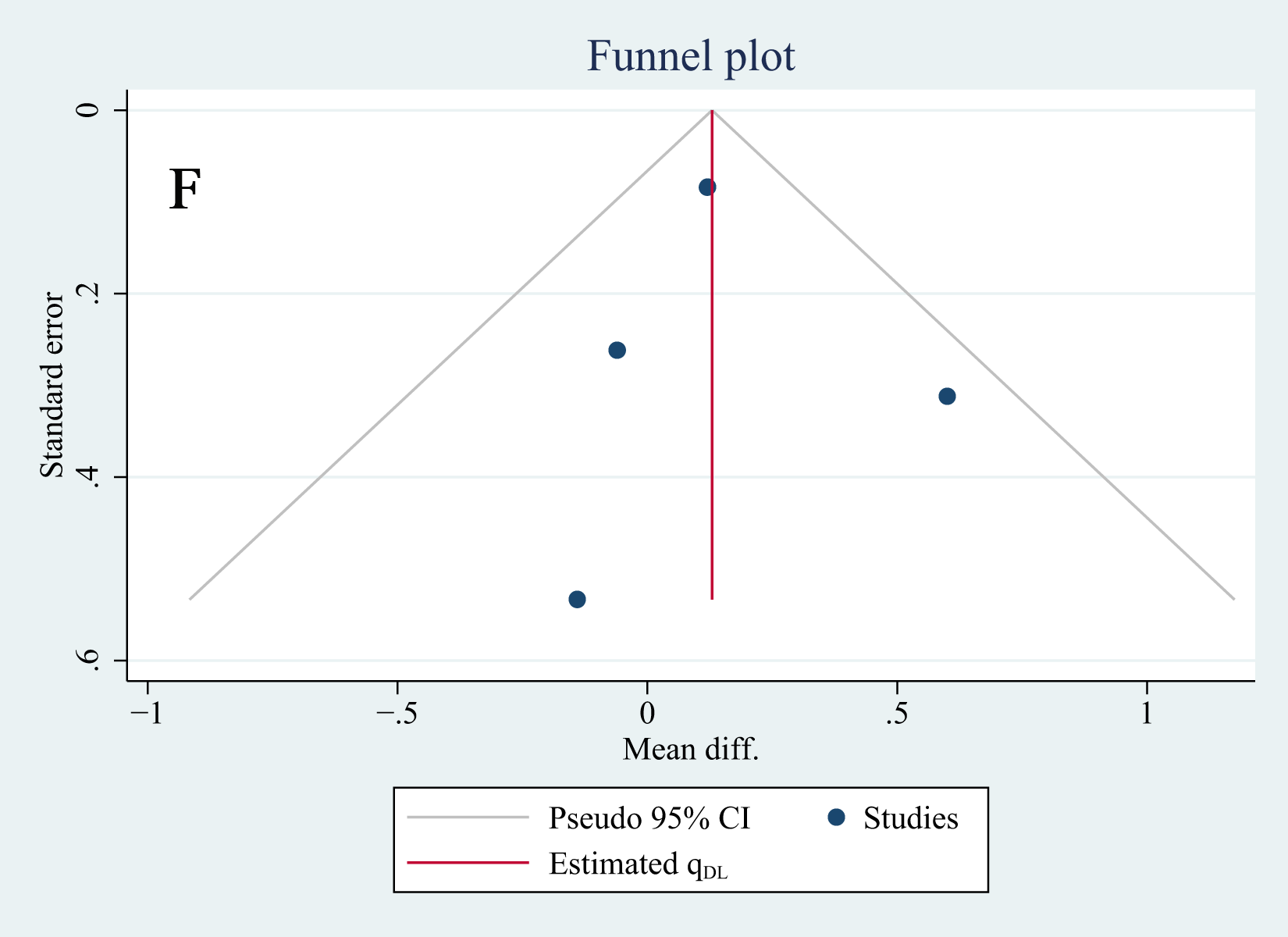


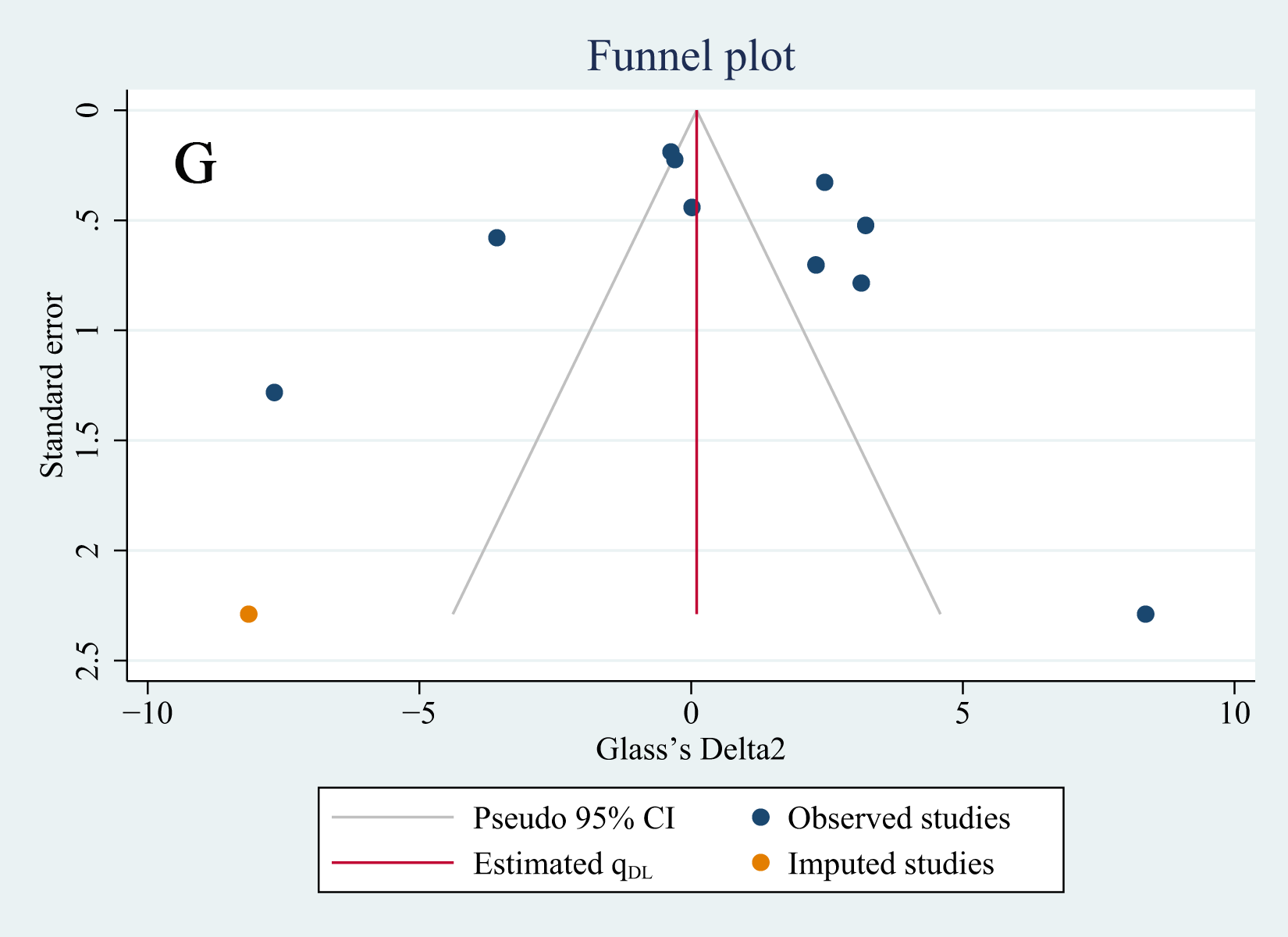


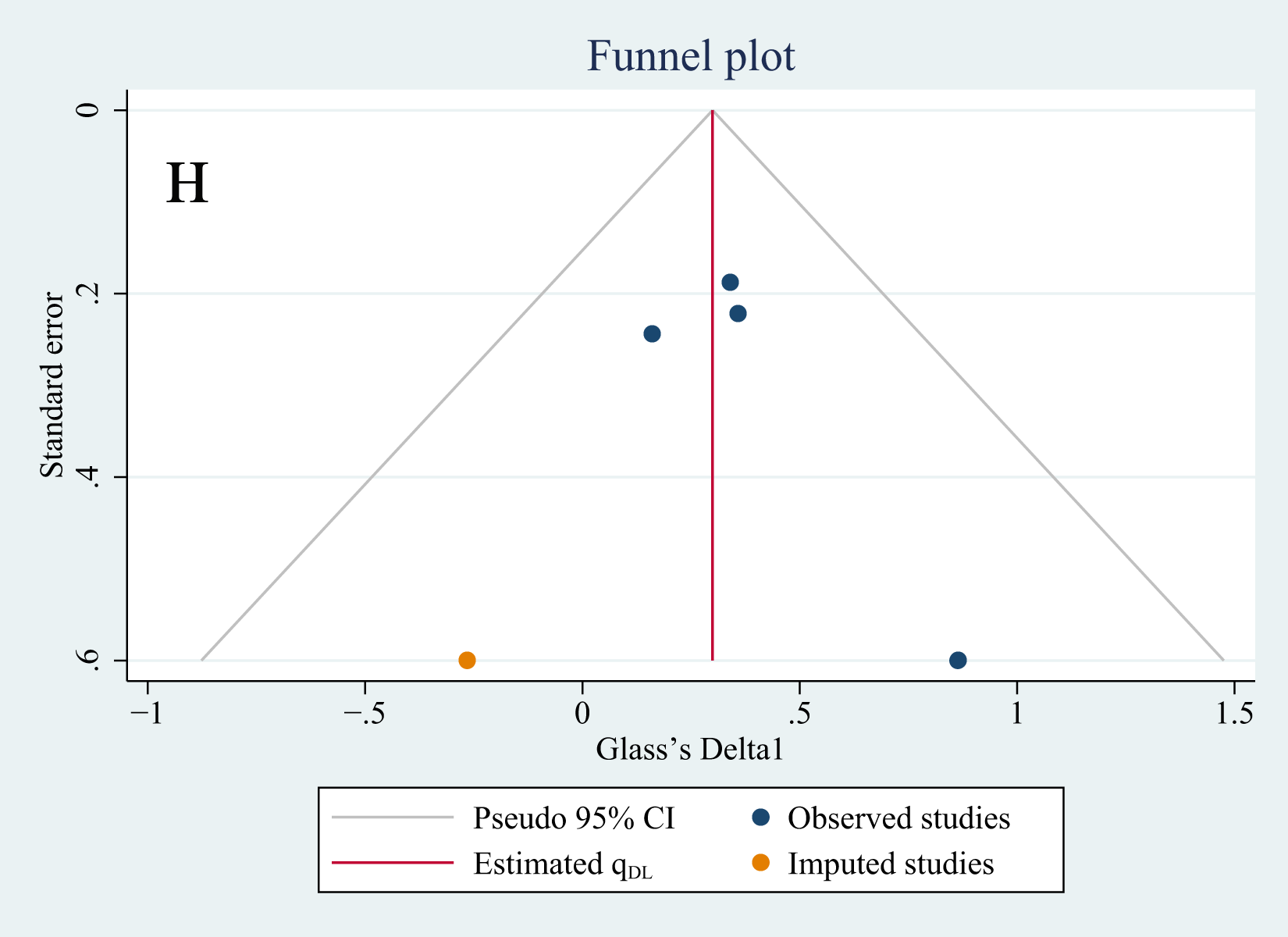


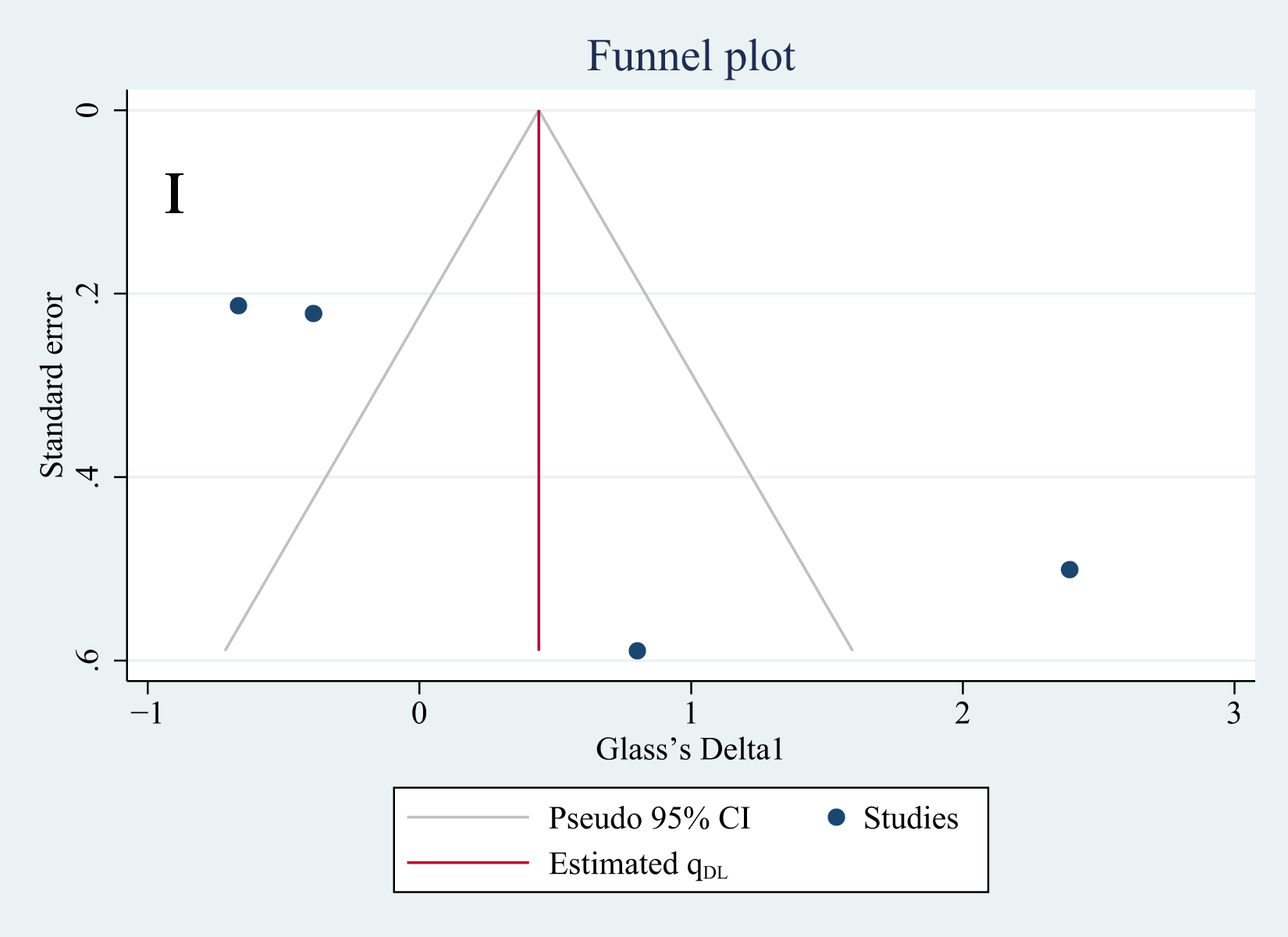


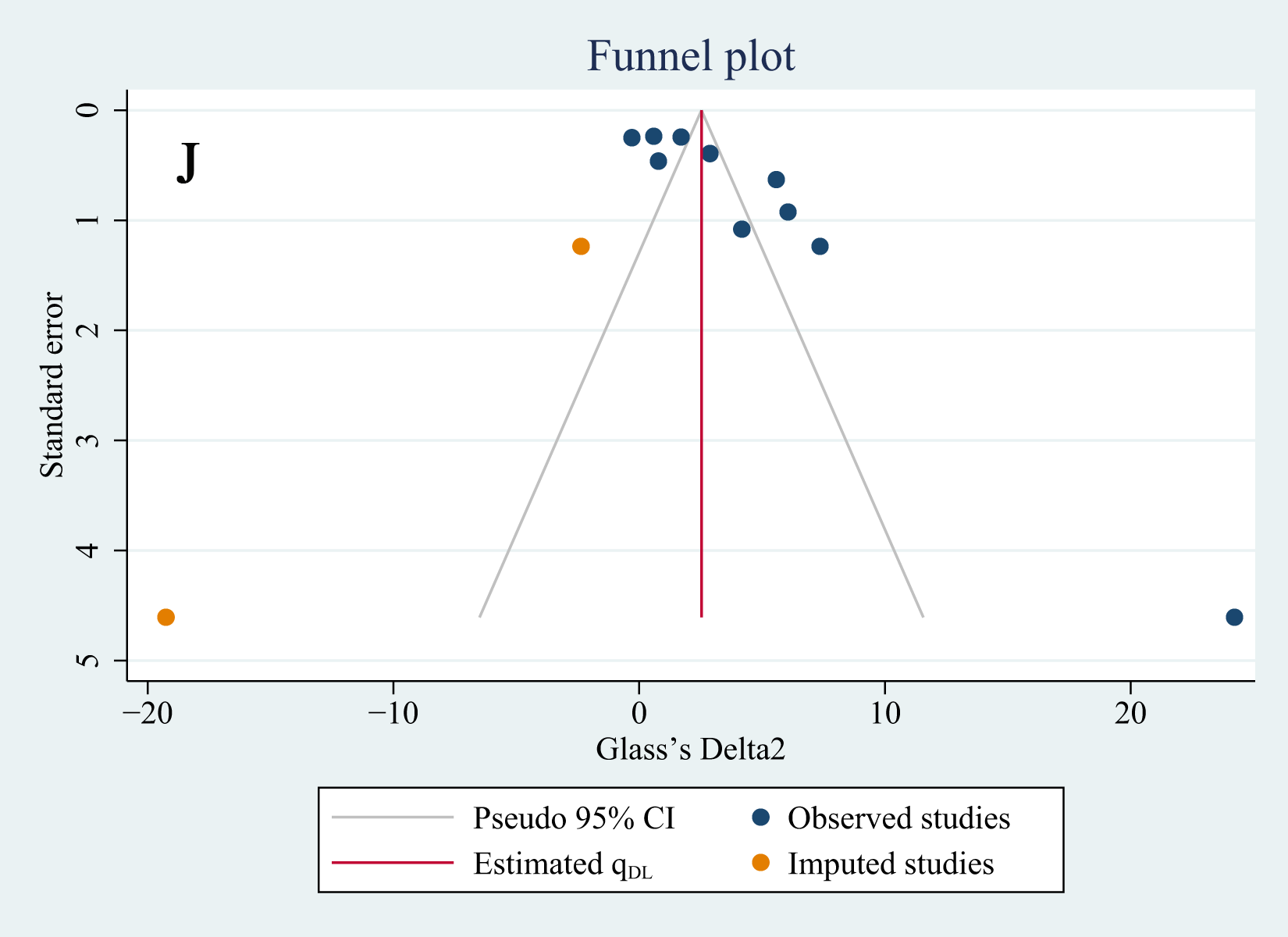


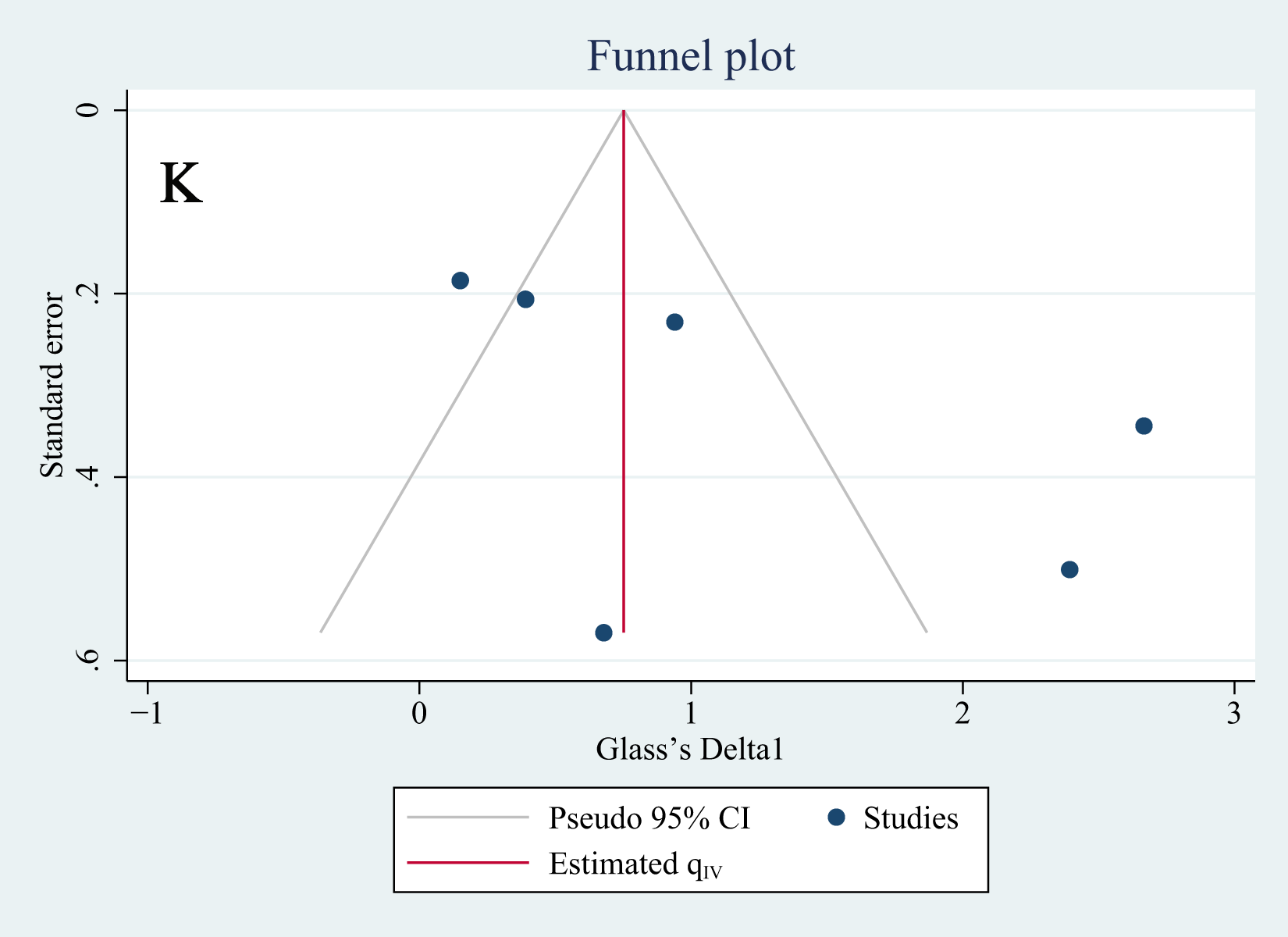


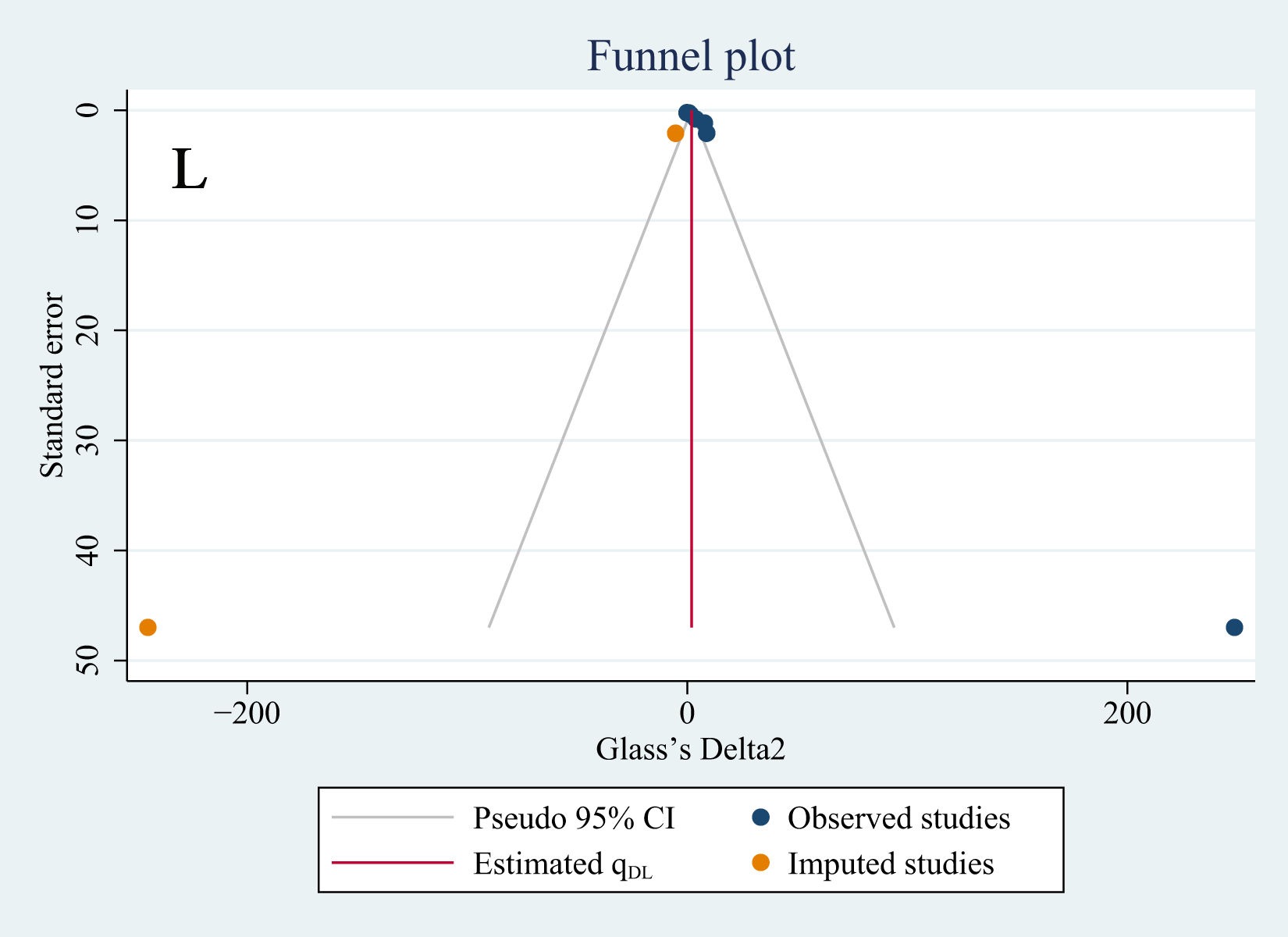


**Supplementary Figure S9.** Funnel chart of the comparison of the Th1, Th2 cells and associated cytokines between SLE patients and HCs. (A) Th1 cell, (B)Th2 cell, (C) Th1 cell (Disease activity), (D) Th2 cell (Disease activity), (E) Th1 cell (kidney injury), (F) Th2 cell (kidney injury), (G) IFN-γ, (H) TNF-α, (I) IL-2, (J) IL-10, (K) IL-6, (L) IL-4.
